# Contactless longitudinal monitoring in the home characterizes aging and Alzheimer's disease–related night‐time behavior and physiology

**DOI:** 10.1002/alz.70758

**Published:** 2025-10-25

**Authors:** Eyal Soreq, Magdalena A. Kolanko, CRT group, Kiran K. G. Ravindran, Ciro della Monica, Victoria Revell, Sarah Daniels, Anna Joffe, Helen Lai, Mara Golemme, Martina Del Giovane, Chloe Walsh, David Wingfield, Ramin Nilforooshan, Marie‐Ange Stefanos, Benjamin Vittrant, Paul de Villèle, Derk‐Jan Dijk, David J. Sharp

**Affiliations:** ^1^ UK Dementia Research Institute Care Research and Technology Centre Imperial College London Sir Michael Uren Hub White City Campus London, and the University of Surrey Guildford UK; ^2^ Department of Brain Sciences Faculty of Medicine Imperial College London Hammersmith Hospital London UK; ^3^ Surrey Sleep Research Centre School of Biosciences University of Surrey Guildford UK; ^4^ Surrey and Borders Partnership NHS Foundation Trust Leatherhead UK; ^5^ Withings Inc. Issy‐les‐Moulineaux France; ^6^ Université Paris Cité NeuroDiderot Inserm Université Paris Cité Paris France

**Keywords:** aging, Alzheimer's disease, bed exits, contactless monitoring, Dementia Research Institute Sleep Index, digital biomarkers, longitudinal study, machine learning, nocturnal behavior, physiological monitoring, sleep disturbances, sleep fragmentation, Withings sleep analyzer

## Abstract

**INTRODUCTION:**

Disturbed sleep patterns are common in dementia but have not been objectively quantified over long periods.

**METHODS:**

We compared a cohort of 83 Alzheimer's disease (AD) patients to 13,588 individuals from the general population. Sleep patterns, heart rate, and breathing rate data were acquired using a zero‐burden contactless, under‐mattress pressure sensor. Data reduction and explainable machine learning approaches were used to identify sleep phenotypes.

**RESULTS:**

AD was characterized by longer time in bed, more bed exits, less snoring, and changes in estimated sleep states. We derived the Dementia Research Institute Sleep Index for Alzheimer's Disease (DRI‐SI‐AD), a digital biomarker quantifying sleep disturbances. DRI‐SI‐AD detected the effects of acute clinical events and dementia progression at the individual level.

**DISCUSSION:**

Our approach may help bridge a gap in dementia care by providing a zero‐burden method for longitudinal monitoring of health events, disease progression, and dementia risk.

**Highlights:**

- Continuous monitoring reveals dementia‐specific nocturnal sleep disturbances.
- We developed a novel sleep biomarker, Dementia Research Institute Sleep Index (AD), for tracking Alzheimer's disease (AD) progression.
- We used contactless under‐mattress sensors for low‐burden, long‐term data collection.
- Prolonged bedtimes and frequent exits were identified as key dementia‐related sleep traits.
- We demonstrated the feasibility of in‐home monitoring for dementia care and risk assessment.

## 1 BACKGROUND

Neurodegenerative disorders causing dementia, such as Alzheimer's disease (AD), commonly cause sleep disturbance,^1^, ^2^ which affects > 65% of patients with AD.^3^, ^4^ In established AD, disruption of sleep patterns is one of the strongest predictors of admission to a nursing home.^5^, ^6^ At the same time, sleep disturbances such as insomnia, sleep‐disordered breathing, sleep fragmentation, and circadian rhythm sleep disorders are associated with a 50% greater risk of developing the disease.^7^

Disturbances of sleep in AD, the most common form of dementia, manifest in various ways. Alterations in sleep physiology include reductions in rapid eye movement (REM) sleep and slow‐wave sleep of non‐REM and associated brain oscillations such as slow waves and sleep spindles.^8^, ^9^, ^10^, ^11^, ^12^, ^13^, ^14^ At the behavioral level, sleep disturbances include difficulties falling or staying asleep, frequent night‐time awakenings, excessive daytime napping, and “sundowning”(i.e. escalations in confusion and agitation during the evening). These behavioral changes are distressing^15^ and often precipitate placements in long‐term care facilities.^6^ Alterations in sleep in dementia are caused by a complex interplay of factors, including neural degeneration in brain regions regulating sleep and circadian cycles; other medical comorbidities; pain and side effects of medications;^16^, ^17^ and environmental factors such as noise, temperature, and light.

Many of these disturbances are also observed in aging,^18^ and to what extent the disturbances in dementia are similar to or exceed those associated with aging has not been quantified in large‐scale studies in the community. Furthermore, our current knowledge about the stability and diversity of sleep disturbance phenotypes and their relation to cognitive status and disease progression has not been measured in continuous longitudinal studies.

These gaps in our knowledge exist because of the difficulty of collecting detailed sleep data over extended periods in people living with or without dementia in at‐home settings. While polysomnography (PSG) provides a wealth of sleep physiology data, it is too cumbersome for longitudinal implementation in the home environment. Importantly, it does not capture aspects of sleep timing and nocturnal interruptions that are of specific relevance to the understanding of the temporal organization of the sleep–wake cycle and its consolidation and are of direct relevance to the caregiver. Questionnaires and diaries rely on patient recall, which becomes increasingly unreliable as dementia progresses and poses a significant burden to the patient and caregiver. Wrist actigraphy, although extensively used in sleep research, suffers from poor patient compliance due to recharging requirements, discomfort with wearing devices, and the burden associated with the requirement to provide additional daily sleep diary information to be considered valid. Consequently, actigraphy data collection periods are usually limited to a few weeks.^19^, ^20^, ^21^ All these limitations have resulted in major questions about the sleep–dementia interrelations remaining unanswered.

We have developed an approach for longitudinal contactless monitoring of sleep disturbances, leveraging ballistographic patterns gathered through the Withings Sleep Analyzer (WSA), a bed‐mat technology. This approach produces a large volume of data, creating challenges for data science. To address this challenge, we have developed a data science framework emphasizing clarity, simplicity, and affordability to make sleep data more interpretable and potentially actionable in real‐world situations. By using explainable (i.e., the ability to assess the model's internal global decision‐making mechanisms) and interpretable (i.e., the ability to assess the model's decision process on an individual basis) machine learning, we facilitate the understanding and application of sleep data in everyday contexts.

We use this framework to address the following questions: (1) How do longitudinal changes in nocturnal sleep behavior among AD patients living at home differ from changes observed in age‐matched older adults? (2) What distinct sleep pattern phenotypes can be identified, and to what extent do these remain stable or exhibit change as individuals age? (3) Are sleep pattern phenotypes associated with AD diagnosis, cognitive abilities, and other measures of the disease state? and (4) Can sleep disturbances be effectively quantified using a single composite sleep disturbance index, facilitating widespread use/implementation?

## 2 METHODS

### 2.1 Overview

This retrospective observational study used data from the WSA, an under‐mattress pressure sensor, to explore the impact of AD on sleep metrics and establish a passive digital biomarker for monitoring sleep disturbances in AD. The study used datasets outlined in the Consolidated Standards of Reporting Trials (CONSORT) diagram (Figure S1 in the supporting information), which details the data processing flow from acquisition and integration to feature engineering, statistical analyses, and model development.

Participants with AD were enrolled in the ongoing prospective longitudinal Minder community‐based cohort study conducted by the UK Dementia Research Institute (DRI), Centre for Care Research & Technology (CR&T). This cohort evaluates at‐home digital technologies to monitor the health of people with dementia and frail elderly adults living at home. The study compared data from AD patients from the Minder cohort to a large general population dataset (≈ 13,500 individuals) provided by Withings.

### 2.2 Recruitment and study setting

The study was conducted in collaboration with Imperial College London and the Surrey and Borders Partnership National Health Service (NHS) Trust. Participants were recruited from memory clinics operated by community mental health teams for older adults and specialist tertiary cognitive clinics in two health‐care trusts: Surrey and Borders Partnership (SABP) NHS Trust and Imperial College Healthcare NHS Trust. Recruitment also occurred through primary care settings, including the Hammersmith and Fulham Primary Care networks.

### 2.3 Participant inclusion and consent

Participants provided written informed consent if they were able to do so. Good Clinical Practice guidelines and the Mental Capacity Act 2005 were followed. If a participant's capacity was reduced, a study partner acted as a consultee representative. Inclusion criteria required participants to be ≥ 50 years old at baseline with a confirmed diagnosis of dementia. Participants and their study partners were required to have sufficient functional English to complete assessment instruments. Exclusion criteria included terminal illness, severe mental health issues, or active suicidal thoughts.

**RESEARCH IN CONTEXT**

1. **Systematic review**: A review of the literature showed that dementia has a marked effect on rest activity and sleep phenotypes, which change through the course of the disease. However, longitudinal, continuous, and objective assessment of rest–activity and sleep patterns in dementia has not been conducted.
2. **Interpretation**: We performed a data science–led analysis of a very long time series of sleep patterns collected via low‐burden contactless technology. The results describe aging and Alzheimer's disease (AD)–specific alterations in sleep behavior and physiology. Long time spent in bed, late arise times, and frequent bed exits is a dominant sleep disturbance phenotype in AD. Within‐patient variations are associated with variation in functional state.
3. **Future directions**: The work demonstrates that information of relevance to dementia can be recorded over long periods from the homes of individuals with and without dementia, providing new information relevant to disease state.

### 2.4 Ethical approvals

The study received approval from the Health Research Authority's London‐Surrey Borders Research Ethics Committee (19/LO/0102). All participants and their study partners provided written informed consent for participation and for their data to be included in publications.

### 2.5 Monitoring equipment

The WSA consists of a thermoplastic polyurethane pad inflated with air connected to a pressure sensor. The unit is covered with a protective sleeve and is powered by a 5 V 1A power supply. A USB power adapter connected to the main socket is included with the unit. Inside the sleeve is the air bladder, which is connected to a case protecting the device's electronic components. The device^22^ is positioned under the mattress, beneath the patient's torso. In brief, the device uses a sensor that measures pressure in the air bladder relative to the atmospheric pressure. The pressure signal is filtered and amplified to isolate three separate mechanical sources: body movements, chest displacement (breathing), and vibrations due to cardiac ejection. They are transmitted by the mattress to the air bladder and are recorded as pressure variations. Data acquisition is initiated when a significant pressure signal is detected. Because significant pressure is associated with bed entry, the data acquisition period provides information on the timing of bed occupancy and bed exits.^23^ The device includes a microphone, which is also placed under the mattress. The pressure and sound signals are analyzed by WSA‐embedded software. Filtered in different frequency bands, the pressure signal provides data on the following physiological functions: breathing, heart rate, and movement. The audio signal is downsampled and filtered to extract a signature of snores.^24^

The WSA approximates sleep states using a combination of advanced sensors and proprietary algorithms. It uses ballistocardiography (BCG), a method that detects the body's movements related to the heartbeat, and a pneumatic sensor to measure respiratory rates and overall body movement. By analyzing these data, the device can distinguish between different sleep stages, including light sleep, deep sleep, and REM sleep. The device uses variations in heart rate, breathing patterns, and physical movement to approximate when a person transitions between these stages. While it does not capture sleep stages with the same precision as full PSG (which includes electroencephalography [EEG] monitoring for brain activity), it provides a practical and non‐intrusive way to monitor sleep patterns and quality at home.^23^, ^24^, ^25^, ^26^ The data collected offers valuable insights into sleep health and helps identify potential sleep disturbances or trends over time.

### 2.6 Data acquisition

As shown in the CONSORT diagram (Figure S1), data were acquired from two primary sources: the Withings general population dataset and the DRI Minder dataset for AD participants. Data for this study were acquired through the Minder platform, a secure digital research platform developed by the UK DRI CR&T at Imperial College London and the University of Surrey, Guildford. The Minder platform integrates various internet‐connected devices and sensors to remotely monitor health and environmental data for people with dementia living in their homes. For this study, participants with AD were enrolled in the ongoing Minder study and provided with a WSA device. The WSA data were securely transmitted to the Minder platform, where it was integrated with other health and environmental data collected from the participants’ homes. The Minder platform implements measures to support privacy and security through encryption and adherence to General Data Protection Regulation (GDPR) regulations. Combining continuous, real‐world sleep data from the WSA and periodic clinical assessments provides a comprehensive dataset for investigating the relationships among sleep patterns, nocturnal behavior, and dementia progression in a naturalistic setting.

### 2.7 Participants and datasets

#### 2.7.1 Withings general population dataset

Data from 13,588 anonymized participants (9942 males) aged between 19 and 100 (64.0 ± 15.7 years) were analyzed and collected between December 2019 and November 2021. All study participants with at least 30 days of WSA data recorded within this period were included in the current analysis. All participants provided written consent through the Withings app for their de‐identified data to be used for research when signing up for a Withings account. The WSA dataset included retrospective period summary aggregates. The dataset consisted of data from 3,786,352 nights. Most of the data (96.7%) were collected in Europe and America, with 75% from five major cities (Berlin 30.2%, Paris 24.5%, London 10.3%, New York 6.9%, and Los Angeles 3.9%). Note that subgroup counts (e.g., by age or sex) may not sum to the total *N* due to the use of a matched sample in subsequent analyses (see CONSORT diagram in Figure S1).

#### 2.7.2 UK DRI Minder dataset

We also analyzed data from 83 participants enrolled in the DRI CR&T Minder study (*N* = 83, 40 males, mean age 82.2 ± 7.7) between January 28, 2019 and January 1, 2025. All participants had an existing clinical diagnosis of AD from either older adult community mental health services or specialist tertiary cognitive neurology clinics. AD was defined by the National Institute of Neurological and Communicative Disorders and Stroke and Alzheimer's Disease and Related Disorders Association (NINCDS‐ADRDA) criteria.^27^

In contrast to the general population data, minute‐by‐minute observations were made available from the AD cohort. This dataset consisted of 29,144,155 minutes across a total number of 52,924 unfiltered nights from 83 ADs, who all had ≥ 60 nights of WSA recordings. Data were recorded when the bed was occupied. Each observation summarizes activity across 1 minute and includes a timestamp indicating the start of the period, heart and respiration rate approximations, a binary feature indicating whether the device captured snoring, and a categorical feature showing the approximation of one of four sleep states (awake, light, deep, and REM).

In addition to the WSA data, the Minder study collected clinical assessments of participants’ cognitive function, neuropsychiatric symptoms, functional performance, and sleep quality using standardized questionnaires administered by trained research staff. Cognitive function was assessed using the Alzheimer's Disease Assessment Scale Cognitive subscale (ADAS‐Cog), providing a quantitative assessment of cognitive domains commonly affected by dementia, including memory, speech, attention, and orientation. Neuropsychiatric symptoms were assessed using the Neuropsychiatric Inventory (NPI), which provides information about domains such as mood, sleep, and appetite. Activities of daily living and functional performance were assessed using the Bristol Activities of Daily Living scale (BADL).^28^ Sleep was assessed using the Pittsburgh Sleep Quality Index (PSQI), providing information about sleep habits and quality over the past month. ADAS‐Cog and PSQI were collected every 6 months, while NPI and BADL were collected every 3 months. All questionnaires were completed face to face by the Minder study research technician, and the questionnaire responses were recorded using a secure Research Electronic Data Capture (REDCap) system.^29^

#### 2.7.3 WSA validation and evaluation

The WSA was validated for use in older individuals and those with dementia in a small population of healthy older adults (*N* = 35; 14 women; mean age: 70.8; standard deviation [SD]: 4.9; range: 65–83 years) and AD (*N* = 11; 5 women; mean age: 72.8; SD: 6.0; range: 61–81 years). In both populations, WSA data were collected for 7 to 14 days at home along with a consensus sleep diary and this was followed by a sleep laboratory assessment. During the laboratory assessment, WSA data were recorded simultaneously with full PSG. The studies were approved by the University of Surrey Ethics Committee (UEC‐2019‐065‐FHMS) and NHS Ethics Committee (22/LO/0694), respectively. A detailed description of the protocol is given in della Monica et al.^30^

The foundational validation of bed occupancy, sleep staging, heart rate, and respiration rate collected via the WSA was conducted in 35 healthy older men and women. In this validation, the performance of the WSA at home was compared to sleep diary and actigraphy (Philips Actiwatch Spectrum), while in the sleep laboratory, the WSA was compared to PSG during a 10 hour time in bed period. The results of this extensive validation have been published elsewhere,^23^, ^26^, ^31^ and we have summarized the core outcomes in the Results section.

We performed an additional validation of the nightly average heart rate and respiration rate of the WSA in a group of 11 AD participants. The WSA nightly average heart rate and respiration rates were compared to PSG electrocardiography (ECG)–derived heart rate and PSG respiratory inductance plethysmography (RIP) thorax‐derived respiration rate using the analysis approach described in della Monica et al.^31^ The results of this validation are provided in the Results section.

### 2.8 Data processing and analysis

This study's data processing, statistical analysis, and visualization were performed using Python (version 3.11.7) and several open‐source libraries. Data manipulation and pre‐processing were performed using Pandas (version 2.2.2) and NumPy (version 1.26.2). Statistical analyses, including descriptive statistics, correlation analysis, hierarchical clustering, propensity score matching, and model development, were conducted using SciPy (version 1.11.4), Statsmodels (version 0.14.0), Scikit‐learn (version 1.5.0), and Pingouin (version 0.5.4). Data visualizations, including plots, charts, and graphs, were created using Matplotlib (version 3.8.0) and Seaborn (version 0.13.2) libraries. Machine learning models were developed using Scikit‐learn (version 1.4.3) and InterpretML (version 0.6.1) for model interpretability. Additionally, the project used various tools and packages, including Black (version 22.12.0), Flake8 (version 6.0.0), Jupyter (version 1.0.0), JupyterLab (version 3.5.3), Kedro (version 0.19.2), and others to ensure a comprehensive and well‐documented workflow.

### 2.9 DRI Sleep Institute feature extraction pipeline

#### 2.9.1 Pre‐processing

Sparse minute‐frequency sleep data were acquired from each household using an application programming interface (API) provided by the sensor manufacturer. To ensure data integrity and consistency, the raw sleep data underwent a pre‐processing step that enforced appropriate data types for subsequent processing steps. To account for variations in patient time zones and to deal with daylight saving periods, the sleep data were localized to align with each patient's local time.

#### 2.9.2 Bed occupancy analysis

Bed occupancy analysis was performed to determine when a patient was in or out of bed. The sleep data were resampled to a 1 minute frequency, and the presence or absence of the patient in bed was determined for each minute. Transitions between bed occupancy states (bed in and bed out) were identified by calculating the differences between consecutive minutes.

#### 2.9.3 Diurnal and nocturnal cluster classification

Bed periods derived from the occupancy transitions were then classified as either diurnal or nocturnal. Bed occupancy periods were first clustered if there was a gap of < 30 minutes. Clusters starting between 8:00 am and 8:00 pm and ending on the same calendar day are considered diurnal. Sleep periods outside this time range or spanning multiple days are classified as nocturnal. These assumptions allowed a general framework for categorizing sleep periods.

#### 2.9.4 Nocturnal sleep metric calculation

Using nocturnal clusters of data, we calculated ≈ 53,000 nightly sleep statistical metrics. These are summarized in Table 1 and include total time spent in bed (in bed time), total time spent out of bed during the nocturnal period (out of bed time), and time spent in each approximated sleep state (e.g., deep sleep, light sleep), derived as the sum of total and state‐specific recorded observations. We also include physiological measures (heart rate, respiratory rate) and total time observed snoring.

**TABLE 1:** Sleep metrics description.

Name	Full name	Description	Unit	Dependency	Formula
Date	Night date	The date calculated as the maximum timestamp of all sleep observations minus 12 hours	%Y‐%m‐%d	datetime	$\mathrm{Date}\;=\max_{i=1}^{k}\left(t_{i}\right)\;-12h$
Time to bed		The first timestamp of all sleep observations for a specific night	%H:%M	datetime	$\;T_{\mathrm{bed}}=\min_{i\;=1}^{k}\left(t_{i}\right)$
Wake‐up time		The last timestamp of all sleep observations for a specific night	%H:%M	datetime	$\;T_{wake}=max_{i=1}^{k}\left(t_{i}\right)$
TO_BED	Going to bed time	Time an individual goes to bed, reflected as angles starting from midday till midday	angles	Time to bed	$\theta \;=\frac{360^{\circ }}{24}\;\times \left(\left(h-12\right)+\frac{m}{60}\right)$
ARISE	ARISE Time	Time an individual wakes up in the morning, reflected as angles starting from midnight until midnight	angles	Wake‐up time	$\theta =\frac{360^{\circ }}{24}\;\times \left(h+\frac{m}{60}\right)$
NOP	Nocturnal occupancy period	Period from going TO_BED to ARISE times	hours	ARISE, TO_BED	$NOP=\frac{ARISE-TOBED}{15^{\circ }}$
IBT	In‐bed time	Total hours spent in bed during NOP	hours	NOP	$IBT=\frac{1}{60}\;\sum_{i\;=1}^{k}1_{t_{i}1\mathrm{NOP}}$
IBP	In‐bed proportion	The ratio of time spent in bed divided by the Nocturnal Occupancy Period	ratio	NOP, IBT	$IBP=\frac{IBT}{NOP}$
OBT	Out‐of‐bed time	Time spent out of bed during NOP	hours	NOP, IBT	OBT=NOP−IBT
EXITS	Bed exits	The number of out of bed episodes observed during the NOP	count	datetime	$EXITS=\sum_{\left\{i=2\right\}}^{k}1_{(t_{i}-\;t_{\left\{i-1\right\}})\;\geq 2}$
EXIT_DUR	Exit duration	Average duration of each exit from bed during the NOP	minutes	OBT, EXITS	$EXI\;T_{DUR}=\frac{OBT*60}{EXITS}$
DEEP_DUR	Deep sleep duration	Total duration of deep sleep estimated by WSA	hours	state	$DEE\;P_{DUR}=\sum_{\left\{i=1\right\}}^{k}1_{\left(\;\mathrm{stat}e_{i}=\;\mathrm{DEEP}\right)}$
AWAKE_DUR	Awake duration	Total duration of awake time estimated by WSA	hours	state	$\;AWAKE_{DUR}=\sum_{\left\{i=1\right\}}^{k}1_{\left(\;\mathrm{stat}e_{i}=AWAKE\right)}$
REM_DUR	REM sleep duration	Total duration of REM sleep estimated by WSA	hours	state	$\;REM_{DUR}=\sum_{\left\{i=1\right\}}^{k}1_{\left(\;\mathrm{stat}e_{i}=\;\mathrm{REM}\right)}$
LIGHT_DUR	Light sleep duration	Total duration of light sleep estimated by WSA	hours	state	$\;LIGHT_{DUR}=\sum_{\left\{i=1\right\}}^{k}1_{\left(\;\mathrm{stat}e_{i}=\;\mathrm{LIGHT}\right)}$
SNT	Snoring duration	Total duration of snoring	minutes	snoring	$SNT=\sum_{\left\{i=1\right\}}^{k}1_{\left(\;s_{i}=\;\mathrm{True}\right)}$
WSA_DEEP	Deep sleep state estimate by WSA	Proportion of total deep sleep divided by total time in bed	ratio	IBT, DEEP_DUR	$WS\;A_{DEEP}=\frac{DEEP_{DUR}}{IBT}$
WSA_AWAKE	Awake period estimate by WSA	Proportion of total awake time divided by total time in bed	ratio	IBT, AWAKE_DUR	$WS\;A_{AWAKE}=\frac{AWAKE_{DUR}}{IBT}$
WSA_REM	REM sleep estimate by WSA	Proportion of total REM sleep divided by total time in bed	ratio	IBT, REM_DUR	$WS\;A_{REM}=\frac{REM_{DUR}}{IBT}$
WSA_LIGHT	Light sleep estimate by WSA	Proportion of total light sleep divided by total time in bed	ratio	IBT, LIGHT_DUR	$WS\;A_{LIGHT}=\frac{LIGHT_{DUR}}{IBT}$
HR	Average nightly heart rate	Average heart rate during the night observations	beats/minute	heart_rate	$HR=\frac{1}{k}\;\sum_{i\;=1}^{k}\mathrm{hear}t_{\mathrm{rat}e_{i}}$
RR	Average nightly respiratory rate	Average respiratory rate during the night observations	cycles/minute	respiratory_rate	$RR=\frac{1}{k}\;\sum_{i\;=1}^{k}\mathrm{respirator}y_{\mathrm{rat}e_{i}}$
SNR	Snoring proportion	Proportion of time spent snoring	ratio	IBT, SNORE_DUR	$SNR=\frac{SNT}{IBT}$

*Notes*: This table summarizes this study's sleep metrics and physiological measures to evaluate nocturnal behavior and sleep patterns in people with AD and age‐matched controls. These metrics were derived from the WSA, a contactless under‐mattress sensor system, and include parameters such as time to bed, wake‐up time, in‐bed time, bed exits, and sleep state distributions. The metrics provide insights into nocturnal occupancy, sleep fragmentation, and physiological states, which are key for identifying sleep disturbances associated with aging and dementia.

Abbreviations: AD, Alzheimer's disease; REM, rapid eye movement, WSA, Withings sleep analyzer.

#### 2.9.5 Feature engineering

The final feature engineering step involved processing the data in the nocturnal sleep clusters identified in the previous stage to generate the night input features used in this study. This stage applied transformations and aggregations to extract relevant sleep metrics from the sparse, non‐uniform time series data. For the sake of this study, WSA sleep summary datasets are treated as clusters, and an identical feature extraction pipeline is applied to them.

Let $S=[s_{1},s_{2},\cdots ,\;s_{k}]$ be a sparse set of sleep observations, where each observation $s_{i}$ has a timestamp $t_{i}$ and represents a set of sleep metrics derived from a minute of observations. The sleep clusters are defined as the time intervals between midday and midday of the next day, allowing for k observations, where k are bounded by 1440 (the number of minutes in a day). Notably, sleep episodes lasting longer than a typical 24 hour circadian cycle do occur and may be more common in AD compared to the general population. However, these prolonged sleep episodes are considered highly unusual and are frequently linked to acute medical events or other exceptional circumstances that fall outside the scope of this study.

The calculated sleep metrics and weighted sleep state averages are concatenated into a single dataset, and any nights with missing values are excluded. The resulting dataset represents the processed DRI Sleep Institute for Alzheimer's Disease (DRI‐SI‐AD) input features used for further analysis and modeling. This stage provides a comprehensive set of sleep metrics and features that capture various aspects of sleep physiology and behavior, including timing, duration, fragmentation, and sleep state composition.

### 2.10 Exploratory and confirmatory data analysis

#### 2.10.1 Data cleaning and pre‐processing

The DRI‐SI‐AD feature dataset was cleaned, and the pre‐processing step was to ensure data integrity and consistency. Sleep metric distributions were then calculated across three age groups from the general population (18–40 years, 40–60 years, and 60–100 years) and those with AD. We computed the lower bound, upper bound, median, and proportion of each sleep metric within each age group and calculated the 2D density mapping of sleep timing (time to bed and wake‐up times) for each age group.

#### 2.10.2 Propensity score matching

To create a balanced sample for model development and ensure unbiased comparisons between AD and the general population, we applied propensity score matching (PSM).^32^ Propensity scores were calculated using logistic regression, with group membership (AD or general population) as the dependent variable and demographic characteristics (age and sex) as predictors. Each AD participant was matched with up to 10 individuals from the general population with the most similar propensity scores, resulting in a matched dataset with equal representation of both groups (MATCHED).

#### 2.10.3 Descriptive statistics and exploratory data analysis

Descriptive statistics were calculated for the engineered features to summarize the characteristics of sleep behavior across age groups and individuals with dementia. The correlation between time to bed and wake‐up time was assessed using circular statistics, and bootstrap confidence intervals were computed. The median and interquartile range (IQR) were reported for all sleep metrics.

#### 2.10.4 Filtering of extreme values

After the exploratory data analysis (EDA) stage, nights with invalid or extreme values were excluded based on predefined criteria. These included limiting the number of bed exits to 20, the in‐bed proportion to 0.6 to 1.0, heart rate between 45 and 85 beats per minute, respiratory rate between 10 and 25 cycles per minute, average exit duration to 60 minutes, out of bed time < 180 minutes; in bed time < 4 hours or > 16 hours were also excluded. People who went to bed before 5:20 pm or after 3:00 am and people who woke up before 2:40 am or after 11:45 pm were also excluded. This was done to allow us to focus on subtle sleep disruptions specific to AD rather than converge on extreme values that were more prevalent in the AD population, as shown by the EDA stage (see CONSORT Diagram in Figure S1).

#### 2.10.5 Comparative analysis across general population and dementia groups

Due to the substantial difference in sample sizes between the groups (i.e., 50,000 vs. 2,000,000 observations), classical statistical comparative analysis was not suitable. The larger sample size of the general population data compared to the dementia group would lead to unequal variances and overpowered tests.^33^ To overcome these issues, we used the Hellinger distance.^34^, ^35^ The Hellinger distance is a type of f‐divergence used to quantify the similarity between two discrete probability distributions. It is a real‐valued function that measures the “closeness” of these distributions. Let $P=(p_{1}\;,p_{2},\cdots ,\;p_{k})$ and $Q=(q_{1}\;,q_{2},\cdots ,\;q_{k})$ be two probability distributions. The Hellinger distance, H(P,Q), between these two distributions is defined as:

$$H\;\left(P,Q\right)=\frac{1}{\sqrt{2}}\;\sqrt{\sum_{i=1}^{k}{\left(\sqrt{p_{i}}-\sqrt{q_{i}}\right)}^{2}}\;$$

Here, *p_i_* and *q_i_* represent the *i*th elements of the distributions *p* and *Q*, respectively. The formula essentially takes the square root of the sum of the squared differences of the square roots of the probabilities and then multiplies it by 1/√2 to normalize the value. This measure was computed between pairs of probability distributions. It encapsulates the differences in the shapes of the distributions, providing a way to evaluate their dissimilarity. This approach is more robust to outliers and less sensitive to sample size imbalance than traditional hypothesis testing. By visualizing the density distributions produced, we investigated the similarities and differences in sleep patterns between the study groups.

### 2.11 Cluster analysis

#### 2.11.1 Sleep–wake state clustering

We used hierarchical agglomerative clustering to identify distinct patterns of bed occupancy, referred to as sleep–wake clusters. The clustering used 15 minute aggregates from the WSA sleep dataset across filtered nights from patients with AD (*N* = 83). Initially, we computed two types of pairwise distances: the Jaccard distance and the Euclidean distance. The Jaccard distance was calculated based on a binary mask of when the bed wasn't occupied, where the metric captures the proportion of non‐overlapping non‐occupied states relative to the total unique states across two nights. This is expressed as: $J\;\left(X,Y\right)=1-|\frac{X\cap Y}{X\cup Y}|$ where |X∩Y| represents the intersection of non‐occupied intervals, and |X∪Y| is the union. The Euclidean distance was computed directly from the 15 minute aggregates representing the number of minutes the bed was occupied to quantify differences in continuous bed occupancy patterns.

These distances were combined into a unified dissimilarity measure using equal weighting. The combined distance matrix was normalized to a similarity matrix in the range [0,1] to enhance interpretability. Hierarchical agglomerative clustering with Ward linkage was applied to this similarity matrix, resulting in six distinct sleep–wake clusters. The optimal number of clusters was determined using the Davies–Bouldin score. To provide a more comprehensive interpretation of each cluster, states were reassigned based on the average duration of bed occupancy across the nights constituting each state, independent of the clustering order.

#### 2.11.2 WSA state classification

To assess our ability to label naïve population data based on nightly aggregates into the identified sleep states, we trained an Explainable Boosting Machine (EBM) classifier.^36^ The classifier was trained on the WSA‐derived variables (IBT, TO_BED, ARISE, NOP, OBT, BED_EXITS, EXIT_DUR) and the sleep state labels derived from the sleep state clustering. The classifier's performance was evaluated using leave‐one‐group‐out^37^ (in which each dementia participant was considered a group) cross‐validation, where the model is iteratively trained on all participants except one and then tested on the held‐out participant. Performance metrics, including precision, F1 score, sensitivity, and accuracy, were computed across all the nights from each held‐out participant. The trained classifier was then used to predict sleep state labels for each night in both the dementia and naïve population datasets.

#### 2.11.3 AD group clustering

Next, we performed a second level of clustering on the sleep state proportions derived for each participant with dementia. The pairwise Hellinger distance between participants’ sleep state proportions was computed and converted into a similarity measure. Hierarchical agglomerative clustering with complete linkage was then applied to these similarities, resulting in four distinct clusters of participants, which we refer to as dementia groups. The optimal number of dementia states was determined to be four based on the silhouette score.

#### 2.11.4 Statistical analysis of cluster effects on outcome variables

To investigate the impact of distinct dementia states on WSA‐derived variables (IBT, TO_BED, ARISE, NOP, OBT, BED_EXITS, EXIT_DUR), we used the Welch analysis of variance (ANOVA) to test for significant differences between states, followed by Games–Howell post hoc tests to identify which specific state pairs differed significantly. For the assessment‐level analysis, we aggregated the predicted sleep states and clinical scale scores (BADL, NPI, ADAS‐Cog, PSQI) for each participant over 90 day windows preceding each clinical assessment. Assessments with < 15 days of WSA data in the preceding 90 days were excluded. The resulting dataset was then clustered using hierarchical agglomerative clustering with Ward linkage, based on the Hellinger distances between the sleep state proportions in each 90 day window. This resulted in four distinct clusters of clinical assessments, which we refer to as assessment states. We then tested the impact of these assessment states on the clinical scale scores using the Welch ANOVA and Games–Howell post hoc tests, as described above. Homoscedasticity was assessed using a Levene test. The significance level (α) was set to 0.05 throughout the analysis, and *p* values were adjusted for multiple comparisons using the Bonferroni correction.

### 2.12 Model development of DRI‐SI‐AD

#### 2.12.1 Model training

An EBM model was trained on a balanced sample from the training data using the selected features and hyperparameters. This gradient‐boosting algorithm provides interpretable explanations for the predictions that are generated. Data from the AD sample (*N* = 83, 40 males, age = 82.2 ± 7.7 years) were compared to age‐ and sex‐matched observations from 790 individuals (400 males, age = 81.3 ± 7.5 years) from the non‐dementia population > 60 years old with at least 180 nights of observations. The trained model's performance was evaluated on the remaining participants who were not included in the bootstrap sample. This iterative fitting process was repeated 1000 times, generating an ensemble of complementary models. The 1000 complementary models obtained from the iterative fitting process were merged using averaging to create the final DRI‐SI‐AD model.

To address group imbalance and reduce overfitting, we used a nested stratified sampling approach with extremely shallow trees on domain expert–selected features, randomly under‐sampled during training. The model's performance was continuously assessed during training using two internal data subsets: a test set to evaluate its ability to generalize to unseen data and a validation set to assess its resistance to overfitting.

#### 2.12.2 Training evaluation

The ensemble model leveraged the diverse models to capture different patterns and relationships in the data, improving overall performance and robustness. Training performance of the DRI‐SI‐AD model's performance was assessed against a dummy model trained on randomly shuffled labels to establish a baseline for comparison. Standard performance metrics were used, including sensitivity, specificity, positive precision, negative precision, Matthew correlation coefficient, F1 score, and area under the receiver operating characteristic curve (AUC‐ROC). This comparison highlighted the DRI‐SI‐AD model's effectiveness in capturing meaningful patterns and relationships during training, demonstrating its validity and reliability in identifying sleep disturbances associated with dementia.

#### 2.12.3 Final model evaluation

The final DRI‐SI‐AD model was further evaluated using the naïve and horizon test datasets to assess the model's discriminative ability and robustness comprehensively. The EBM consistently outperformed the dummy model across all performance metrics and data subsets, demonstrating robustness and reliability.

#### 2.12.4 Post hoc permutation importance test

To assess the feature importance, we used permutation importance, a model‐agnostic approach performed after model training. This method involves randomly shuffling the values of each feature and measuring the impact on the model's performance. The rationale is that if shuffling a feature's values significantly decreases model accuracy, the feature is considered important. Specifically, the procedure involves assessing the impact of removing the informative value of each feature and assessing the relative drop in the F1 score as a function of that shuffle.

Features that caused a significant drop in model performance when permuted were deemed crucial for accurate predictions. In our study, key features such as bed exits and exit duration were found to be highly influential in distinguishing between AD and the control group. This aligns with previous findings that highlighted the importance of nocturnal activity patterns in dementia assessment.

#### 2.12.5 Multi‐level splitting strategy

We used a multi‐level splitting strategy to evaluate the DRI‐SI‐AD model's performance.^38^ First, 10% of participants (8 AD and 81 individuals from the general population) were excluded to serve as a naïve test dataset, allowing for an independent assessment of the model's generalization ability. From the remaining 90% of the data, the last 30 days of observations were used to create a naïve horizon test dataset, enabling evaluation of the model's performance on future, unseen data points.

#### 2.12.6 Iterative fitting approach

To robustly estimate the DRI‐SI‐AD model's performance and account for variability in the data, we used an iterative fitting approach using bootstrapping, quasi‐random temporal sampling, and random under‐sampling techniques.^39^ The process involved the following steps:

1. Bootstrap sampling: A stratified bootstrap sample of participants was drawn from the matched dataset, preserving the balanced representation of AD and general population.
2. Quasi‐random temporal sampling: Within each bootstrap sample, quasi‐random temporal sampling was applied to select observations for model training, ensuring exposure to diverse temporal patterns while maintaining the temporal order of observations.
3. Random under‐sampling: Random under‐sampling was used to address class imbalance within each training set by randomly removing observations from the majority class (general population) to achieve a balanced class distribution.

### 2.13 Clinical cases and disease progression

To illustrate the diverse manifestations of the DRI‐SI‐AD over extended periods, we also conducted a retrospective analysis of the Minder study records corroborated by the health‐care records from two participants with progressing DRI‐SI‐AD trajectories. After identifying the cases, a clinician manually reviewed the study records, and participants’ health‐care records and medication records were also accessed to identify any clinical or environmental factors that may have contributed to the abnormal DRI‐SI‐AD trajectories. Case reports were structured to concisely describe each participant's unique dementia manifestation, the DRI‐SI‐AD trajectory, and notable events or changes in their clinical status. To investigate the relationship between the DRI‐SI‐AD and dementia progression, we examined longitudinal data from our AD participants were assessed using standard clinical scales for disease progression every 3 months (NPI and BADL) or every 6 months (PSQI and ADAS‐Cog) over 18 months, resulting in a total of 1400 completed assessments.

#### 2.13.1 Analysis of association between DRI‐SI‐AD, DRI‐SI‐AD variability, and clinical scores using mixed‐effects linear model

To examine the association between the DRI‐SI‐AD and its variability (DRI‐SI‐AD_VAR) with clinical scores while controlling for several covariates, we used a mixed‐effects linear model. The analysis was performed using the smf.mixedlm function from the statsmodels library in Python. This model allows us to account for both fixed and random effects, thus accommodating the repeated measures and hierarchical structure of our data.

The mixed‐effects linear model is specified as follows:

$$\begin{array}{ccc} \mathrm{SCOR}E_{\mathrm{ij}} & = & \beta_{0}+\beta_{1}\;\hat{\mu }{\left(\mathrm{DRI}-\mathrm{SI}-\mathrm{AD}\right)}_{\mathrm{ij}}+\beta_{2}\;{\hat{\sigma }}^{2}{\left(\mathrm{DRI}-\mathrm{SI}-\mathrm{AD}\right)}_{\mathrm{ij}}\\ & & +\;\beta_{4}\;\mathrm{AG}E_{\mathrm{ij}}+\beta_{5}\;\mathrm{SE}X_{\mathrm{ij}}+u_{i}+\varepsilon_{\mathrm{ij}} \end{array}$$

with the patient id acting as random effect $u_{i}$.

## 3 RESULTS

### 3.1 WSA accurately records sleep, behavior and physiology patterns

**FIGURE 1:**
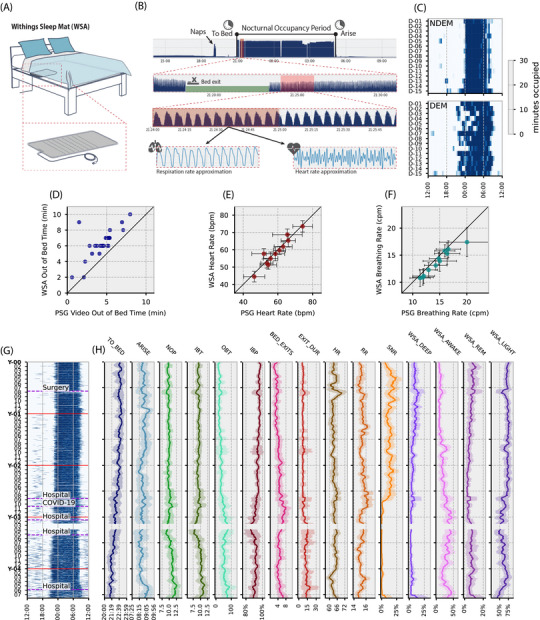
WSA capabilities and study design. A, WSA, an under‐mattress sensor system. B, Raw occupancy signal and the derivation of TO_BED and ARISE times and the presence of a nap in the early evening (top panel). Higher resolution data with missing signal due to a bed exit (second panel). Respiratory and heart rate approximation (last three panels). C, Raster plots showing bed occupancy over a 14 day period from patient with and without dementia. D‐F, Scatter plots showing relationship between laboratory PSG and WSA for out of bed time, heart rate (beats per minute), and breathing rate (cycles per minute). G, Illustration of how longitudinal data collected over different periods has the potential to provide clinically relevant information of different types. Data from a representative individual with Alzheimer's disease showing fluctuations in bed mat occupancy over 4.5 years. G, The heatmap represents days in the *y* axis and time in the *x* axis with blue color indicating bed occupancy. H, Nightly features derived from the minute‐by‐minute information provided by WSA, each line represents a rolling mean (to reduce local noise) of a month, and the shaded area represents the rolling standard variation (see Table 1 for feature descriptions and units). ARISE, time leaving bed; BED_EXITS, number of bed exits; DEM, dementia; EXIT_DUR, length of time out of bed; HR, heart rate; IBP, in‐bed proportion; IBT, in‐bed time; NDEM, without dementia; NOP, nocturnal occupancy period; OBT, out‐of‐bed time; PSG, polysomnography; RR, respiration rate; SNR, snoring; TO_BED, time to bed; WSA, Withings sleep analyzer; WSA_AWAKE, time awake estimate by Withings sleep analyzer; WSA_DEEP, deep sleep state estimate by Withings sleep analyzer; WSA_LIGHT, light sleep state estimate by Withings sleep analyzer; WSA_REM, rapid eye movement sleep estimate by Withings sleep analyzer.

We used the WSA as an unobtrusive contactless under‐mattress sensor system that allows continuous long‐term monitoring (Figure 1A). The WSA records BCG data and audio signals during sleep at 250hHz (Figure 1B). This is converted to a minute‐by‐minute dataset. From this, sleep measures can be derived, and previously, several studies have evaluated Withings measures against gold standard measures, primarily in young and middle‐aged participants.^22^, ^24^ Here, we evaluated WSA against gold‐standard measures in older adults and people living with dementia (PLWD) using a protocol that collected measures over 7 to 14 days at home (e.g. Figure 1C), followed by a sleep laboratory assessment (Figure 1D‐F and S2A,E in supporting information). Based on these validation studies, we defined and selected variables to describe sleep patterns in AD (Figure 1G,H and Table 1).^23^, ^26^

The WSA showed high correspondence with reference measures for bed occupancy and bed exits^23^ (Figure S2B‐G). In older people, WSA‐estimated time to bed (TO_BED) and time leaving bed (ARISE) times at home highly correlated with those assessed by the daily consensus sleep diary (*n* = 27; nights = 306; TO_BED: *r* = 0.96 [0.951, 0.968], *p* < 0.001; ARISE: *r* = 0.99 [0.989, 0.993], *p* < 0.001).^23^ The WSA also accurately recorded ≈ 90% of daytime naps in bed in older people.^23^

WSA‐estimated duration of bed exits strongly correlated with estimates from manual video inspection in the sleep laboratory for both older participants without dementia (*n* = 35; events = 30, *r* = 0.891 [0.731, 0.958], *p* < 0.001)^23^ and AD (*n* = 11; events = 26, *r* = 0.724 [0.362, 0.896], *p* < 0.001). Nightly average heart rate and respiratory rate from the WSA strongly correlated with PSG measures in older participants (*n* = 35; *r* = 0.877 [0.768, 0.936], *p* < 0.001 and *r* = 0.778 [0.601, 0.882], *p* < 0.001 for heart rate and breathing rate, respectively^31^) and AD (*n* = 11; *r* = 0.952 [0.822, 0.988], *p* < 0.001 and *r* = 0.971 [0.888, 0.993], *p* < 0.001 for heart rate and breathing rate, respectively).^31^ WSA‐estimated snore duration also correlated well with the PSG estimate (*n* = 30; *r* ^2^ = 0.76, *p* < 0.001).^31^ However, in older participants, WSA estimates of light, deep, and REM sleep states did not significantly correlate with PSG‐determined sleep stages (WSA_light_ vs. N1/N2: *r* = –0.555 [–0.752, –0.268], *p* < 0.001; WSA_Deep_ vs. N3: *r* = 0.165 [–0.184, 0.476], *p* = 0.35; WSA REM vs. REM: *r* = 0.052 [–0.291, 0.384], *p* = 0.769^26^). In our larger AD cohort, caregiver‐reported PSQI estimates of sleep time correlated with WSA‐estimated in‐bed time (IBT; *r* = 0.48, *p* < 0.0001). TO_BED and ARISE times also significantly correlated with reported bedtime and wake time from PSQI (*r* = 0.72, *p* < 0.0001 and *r* = 0.69, *p* < 0.0001, respectively; Figure S3 in supporting information).

Based on these evaluation studies, we analyzed TO_BED and ARISE times and the total time spent in bed, that is, the nocturnal occupancy period (NOP). Within the NOP, we calculate the IBT and the out‐of‐bed time (OBT), as well as the in‐bed proportion (IBP)—the ratio of time spent in bed to the total NOP. We also determined the number of times an individual leaves the bed (BED_EXITS) and their duration (EXIT_DURATION). Additionally, we analyzed the average nightly heart rate (HR), respiratory rate (RR), and snoring rate (SNR). Although in older participants the WSA provides a weak estimate of sleep stages,^26^ these measures may provide some information about vigilance states. We therefore report these as WSA_AWAKE_, WSA_REM_, and for non‐REM sleep, WSA_LIGHT_ and WSA_DEEP_.

The ability to measure nightly for long periods of time offers a scalable way to investigate changes in sleep patterns seen as neurodegenerative disease progresses, which we evaluate in subsequent sections. This is illustrated in data from an 81‐year‐old patient with AD studied over > 4 years. Figure 1G shows all these variables derived from WSA data collected during this period. The data show fluctuations superimposed on progressive longitudinal changes for many of these variables. For example, over years TO_BED time becomes earlier, ARISE time become later, and snoring almost completely vanishes as the disease progresses.

### 3.2 Differences in longitudinal changes of nocturnal sleep between AD patients and age‐matched controls

**FIGURE 2:**
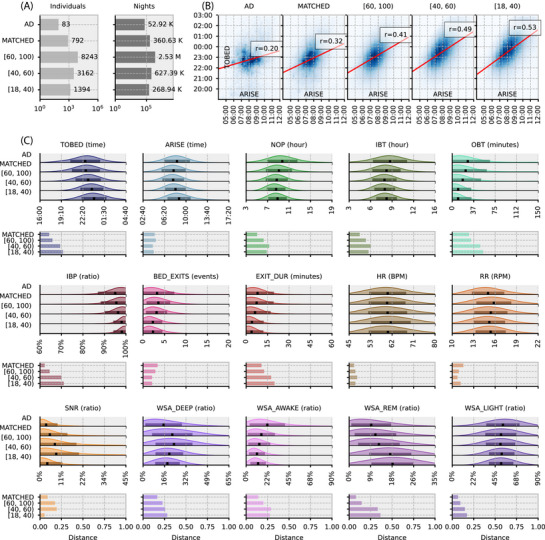
Night‐time behavior, physiology, and sleep in AD compared to the general population: (A) bar plots showing counts of individuals and nights across age bands and groups, (B) scatter plot of ARISE and TO_BED times across the age bands with association lines calculated using circular statistics, (C) normalized density distributions of core bed metrics across age bands. The dark line reflects the mean per age band, and the shaded area reflects ± one standard deviation of the distribution around the mean. Each metric is accompanied by relative distance bar plots showing how similar age bands are to the AD group using Hellinger distance (larger values indicate greater dissimilarity; see Methods section). The distributions show that participants with AD have longer IBP, longer IBT, more BED_EXITs, and increased EXIT_DUR. (see Table 1 for feature descriptions). AD, Alzheimer's disease; ARISE, time leaving bed; BED_EXITS, number of bed exits; EXIT_DUR, length of time out of bed; HR, heart rate; IBP, in‐bed proportion; IBT, in‐bed time; NOP, nocturnal occupancy period; OBT, out‐of‐bed time; RR, respiration rate; SNR, snoring; TO_BED, time to bed; WSA, Withings sleep analyzer; WSA_AWAKE, time awake estimate by Withings sleep analyzer; WSA_DEEP, deep sleep state estimate by Withings sleep analyzer; WSA_LIGHT, light sleep state estimate by Withings sleep analyzer; WSA_REM, rapid eye movement sleep estimate by Withings sleep analyzer.

We compared sleep and night‐time behavior (Figure 2A) in the general population (3,786,352 nights; *N*_population _= 13,588, 9942 males, mean age 67.5 ± 14.2 years; range: 19–100) and in patients living with AD (*N*_AD _= 83, 40 males, mean age 83.2 ± 7.8; range 60.9–94.8). A propensity score‐matched group (MATCHED) was generated from the general population (*N*_MATCHED _= 779, 400 males, mean age 82.6 ± 6.8; range 60.0–98.8 years) matched by age and sex to the AD group (Figure 2 and Table S1 in supporting information).

TO_BED and ARISE times varied with age and disease state (Figure 2C). TO_BED time became earlier with age, accompanied by an earlier ARISE time. The matched adults had the earliest TO_BED (22:44 ± 2.2 hours), while the youngest group had the latest (23:59 ± 1.9 hours). The 60 to 100 age group had the earliest ARISE time (07:52 ± 2.5 hours), and the 18 to 40 age group had the latest (08:50 ± 2.1 hours). IBT remained relatively stable across age groups but was shortest in the 40 to 60 age group (8.6 ± 2.1 hours). In contrast, AD had both early TO_BED (22:47 ± 2.8 hours) and late ARISE times (08:29 ± 2.4 hours), resulting in increased IBT (9.3 ± 3.1 hours).

Across the whole population, TO_BED was strongly correlated with ARISE times (Figure 2B). This association became less marked as individuals aged and almost non‐existent in the AD population (*r*_AD_ = 0.2 [95% confidence interval [CI] 0.10, 0.24], *r*_MATCHED_ = 0.32 [95% CI 0.29, 0.43], *r*_[60, 100)_ = 0.41 [95% CI 0.37, 0.49], *r*_[40, 60)_ = 0.49 [95% CI 0.48, 0.59], r _[18, 40)_ = 0.53 [95% CI 0.58, 0.67]).

AD had the longest average time out of bed (33 ± 62.9 minutes), with bed exits occurring on 83% of nights, while the youngest group had the shortest (11 ± 41.0 minutes), with at least one bed exit recorded on 70% of nights. Both AD (3.5 ± 5.5 events), MATCHED (3.6 ± 2.9 events), and the 60 to 100 age group (3.1 ± 2.5 events) exited their bed more during the night than the two youngest groups (2.3 ± 2.1 events). Bed exit durations were the longest in AD (9.6 ± 23.9 minutes); however, in older adults, exit durations were still longer (8.7 ± 26.5 minutes) than those of the 18 to 40 age group (4.3 ± 17.3 minutes).

HR and RR were consistent across age groups and individuals with dementia. However, a 60 beats per minute peak was observed in a small number of patients with active pacemakers. Snoring increased with age and was most common in the 40 to 60 and 60 to 100 groups. However, snoring was less common in AD, returning to levels like that seen in the 18 to 40 age group.

Age and dementia also affected vigilance states, as estimated by the WSA. The time spent in deep sleep estimated by WSA_DEEP increased with age and was highest in the 60 to 100 age group (23.4% ± 13.8), but was lowest in AD (15.5% ± 13.7). In contrast, AD participants spent the most time in WSA_AWAKE_ state (22.3% ± 18.3) with the 40 to 60 group spending the least time (11.4% ± 8.1). Individuals with AD spent the least amount of time in WSA_REM_ state (9.0% ± 8.2), with the youngest group spending the most amount of time (17.7% ± 8.7). Finally, WSA_LIGHT_ state remained the same across the groups; however, the variance was higher in the older groups (53.2% ± 17.3), compared to the young (51.3% ± 12.6 see Table S1).

### 3.3 Identification of AD sleep pattern phenotypes across age

**FIGURE 3:**
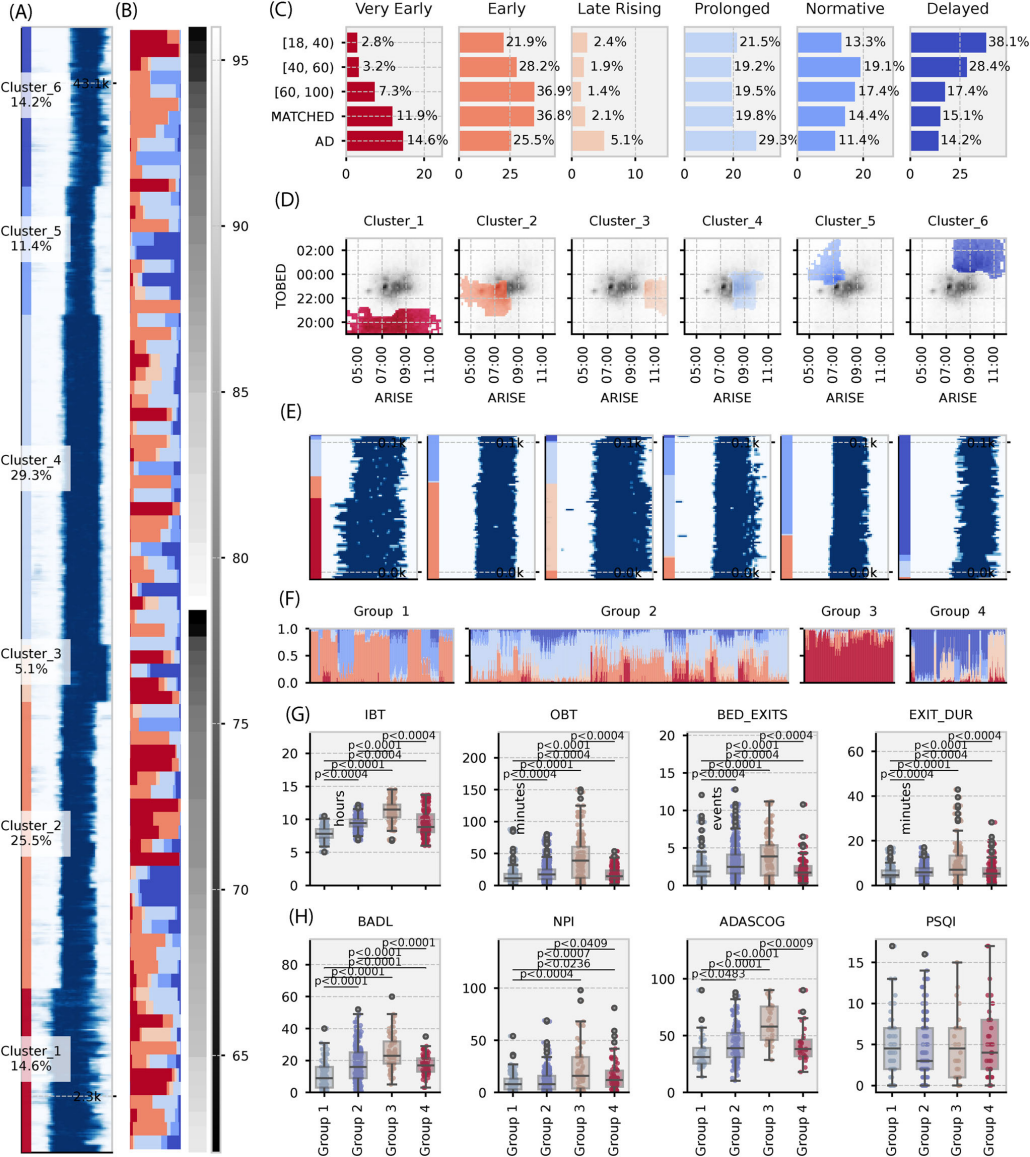
Cluster analysis of bed occupancy patterns identifying distinct sleep–wake profiles in AD patients compared to the general population. A, Raster plot illustrating time spent in bed (dark blue) and out of bed (white) across ≈ 43,000 nights in AD patients, grouped into six identified clusters. Rows represent individual nights, spanning from noon to noon (1440 minutes), and are sorted by cluster assignment. B, Grid plot showing the cluster membership proportions for each AD patient. Rows = individual patients, ordered vertically by sex (females at the top, males at the bottom) and age 60 to 100 years old. C, Bar charts summarizing the proportion of nights assigned to each sleep–wake cluster in AD patients, matched controls, and general population age groups (18–40, 40–60, 60–100 years). Each chart is color‐coded according to cluster assignment. D, 2D density plots showing the distribution of TO_BED (*y* axis) and ARISE (*x* axis) times for each cluster. AD data (colored by cluster) are overlaid on the general population distribution (gray, as shown in Figure 2B) for comparison. E, Six exemplar raster plots showing nightly sleep–wake cluster assignments across consecutive 90 day periods for selected AD patients, highlighting intra‐individual variability. Days are sorted by cluster assignment. F, Stacked bar plots showing the distribution of sleep–wake clusters within 1350 distinct 90 day periods preceding clinical assessments. Each vertical bar represents a single patient‐period, sorted horizontally by their assigned clinical group (groups 1–4). Vertical color segments indicate nightly cluster assignments, ordered by cluster (not time), to emphasize composition rather than temporal sequence. Clinical assessments occurred at the top of each bar. G, Boxplots comparing sleep metrics—IBT, OBT, BED_EXITS, and EXIT_DUR—across clinical groups, annotated with post hoc significance tests. H, Boxplots comparing clinical outcomes, including functional impairment (BADL), neuropsychiatric symptoms (NPI), cognitive performance (ADAS‐Cog), and subjective sleep quality (PSQI), showing significant differences across the four groups. AD, Alzheimer's disease; ADAS‐Cog, Alzheimer's Disease Assessment Scale Cognitive subscale; ARISE, time leaving bed; BADL, Bristol Activities of Daily Living; BED_EXITS, number of bed exits; EXIT_DUR, length of time out of bed; IBT, in‐bed time; NOP, nocturnal occupancy period; NPI, Neuropsychiatric Inventory; OBT, out‐of‐bed time; PSQI, Pittsburgh Sleep Quality Index; TO_BED, time to bed.

Discovery proceeded in three steps. Step 1 (night level, AD only): we clustered all nights pooled across all AD participants (*n* = 83, nights = 45,398, Figure 3A), ignoring patient identity. This used a night‐to‐night similarity matrix and yielded six bed‐occupancy patterns (Figure 3A–B). Step 2 (external characterization): we trained a multi‐class classifier on these patterns and applied it to the normative dataset (3,400,000 nights) to test age associations in cluster prevalence (Figure 3C). Step 3 (pre‐scale 90 days): for each 90 day window preceding a clinic visit, we computed the proportion of nights assigned to each of the six patterns and re‐clustered these proportion vectors, producing four higher level sleep‐occupancy groups used in the clinical comparisons (Figure 3D–E). Membership is per window and time varying: 56/77 (≈ 73%) patients remained in one group across 1015 windows, 20/77 (≈ 26%) alternated between two groups across 376 windows, and 1/77 (≈ 1%) occupied multiple groups across 22 windows (total 1413 windows) (Figure 3F).

#### 3.3.1 Patient‐independent clustering of AD nights identifies six patterns

Step 1 was night‐level discovery (AD nights, patient identity ignored). Using a night‐to‐night similarity matrix over WSA features (TO_BED, ARISE, IBT, OBT, BED_EXITS, EXIT_DUR), yielded six distinct bed‐occupancy patterns. For illustration: very early, fragmented (cluster 1) showed the longest IBT (11.8 ± 1.9 hours), very early TO_BED (19:30 ± 56 minutes), and typical ARISE (07:50 ± 1 hour 49 minutes); prolonged (cluster 4) had moderate IBT (9.7 ± 0.9 hours), average TO_BED (23:00 ± 42 minutes), and late ARISE (09:03 ± 34 minutes); normative (cluster 5) had the shortest IBT (6.8 ± 1.0 hour), late TO_BED (00:05 ± 44 minutes), and early ARISE (07:04 ± 36 minutes). Full descriptives are in Table S2 in supporting information.

#### 3.3.2 Age‐related differences in sleep clusters

Step 2 was external characterization (normative dataset). A multi‐class classifier trained to label these six patterns achieved macro‐F1 = 0.90 ± 0.11, precision = 0.92 ± 0.08, sensitivity = 0.91 ± 0.10 (per‐participant results in Table S3 in supporting information). Applied to 3,400,000 normative nights, cluster prevalence differed between AD and all control age bands ([MATCHED] χ^2^ = 58.77; [60–100 years] χ^2^ = 101.00; [40–60 years] χ^2^ = 176.15; [18–40 years] χ^2^ = 214.40; all *p* < 0.001). The MATCHED cohort most closely resembled AD, whereas the youngest controls differed most (Figure 3C). Cluster prevalence showed clear age gradients. Very early, fragmented (cluster 1), late rising, disrupted (cluster 3), and prolonged (cluster 4) increased with age and were over‐represented in the AD group relative to controls (standardized residuals |*r*| > 2.58, *p* < 0.01). In contrast, normative (cluster 5) and delayed bed timing (cluster 6) were most common in younger/middle‐aged controls and declined with age, with the steepest decline in AD (Figure 3D).

#### 3.3.3 Comparison of AD to MATCHED controls

Directly comparing AD to the MATCHED control group, distinct differences in bed occupancy patterns were observed. Very early, fragmented bed occupancy (cluster 1), Late Rising, disrupted bed occupancy (cluster 3), and prolonged bed occupancy (cluster 4) were significantly over‐represented in AD. Conversely, early to bed and rise (cluster 2) was notably under‐represented (residual = –3.21, *p* < 0.01). No significant differences were observed for normative bed occupancy (cluster 5) and delayed bed timing (cluster 6).

### 3.4 Clinical associations of 90 day sleep‐occupancy groups in AD

In Step 3 (pre‐scale 90 days), we examined associations between bed‐occupancy clusters and clinical measures using data from 77 patients across 1413 observation windows, each summarizing the 90 days preceding a clinic assessment. Clusters were not static; many patients shifted cluster even within a 90 day window (illustrated in six exemplar cases; Figure 3E). To avoid circularity, we then re‐derived clusters from sleep‐occupancy features alone (independent of clinical measures), yielding four groups: (1) normative bed occupancy (*n* = 20; 313 periods), (2) prolonged bed occupancy (*n* = 48; 695 periods), (3) fragmented bed occupancy (*n* = 16; 195 periods), and (4) delayed bed timing (*n* = 15; 209 periods). Membership was time varying rather than fixed: 56 patients remained in a single state across 1015 periods; 20 patients occupied two states across 376 periods; and 1 patient occupied three states across 22 periods. Thus, cluster assignment is neither fixed nor mutually exclusive and can shift over time. Subsequent comparison of these groups with independent clinical assessments revealed differing functional and cognitive impairment levels. A Welch ANOVA identified significant main effects across multiple behavioral metrics (Figure 3G) and clinical measures (Figure 3H), except PSQI (*p* < 0.001). Post hoc Games–Howell comparisons demonstrated specific differences: age varied significantly (*F*[3,554] = 35.27, *p* < 0.001), with cluster 3 (fragmented bed occupancy) being notably older *(g* = 0.790, *p* < 0.0001). Groups differed significantly in WSA features, including IBT (*F*[3,452] = 281.4, η^2^ = 0.37), OBT (*F*[3,506] = 40.36, η^2^ = 0.171), BED_EXITS (*F*[3,519] = 22.6, η^2^ = 0.05), and EXITDUR (*F*[3,441] = 12, η^2^ = 0.078), with cluster 3 exhibiting more pronounced impairment (Figure 3G).

Clinically, cluster 3 showed significantly greater functional impairment (BADL: *F*[3,481] = 30.04, η^2^ = 0.157), increased neuropsychiatric symptoms (NPI: *F*[3,485] = 15.3, η^2^ = 0.09), and reduced cognitive function (ADAS‐Cog: *F*[3,210] = 14, η^2^ = 0.17; Figure 3H). These results align with existing literature highlighting sleep disturbances in dementia and AD, notably early bedtime, prolonged time in bed, and fragmented sleep continuity (e.g., Balouch et al.^15^).

### 3.5 Development and evaluation of the DRI‐SI‐AD

**FIGURE 4:**
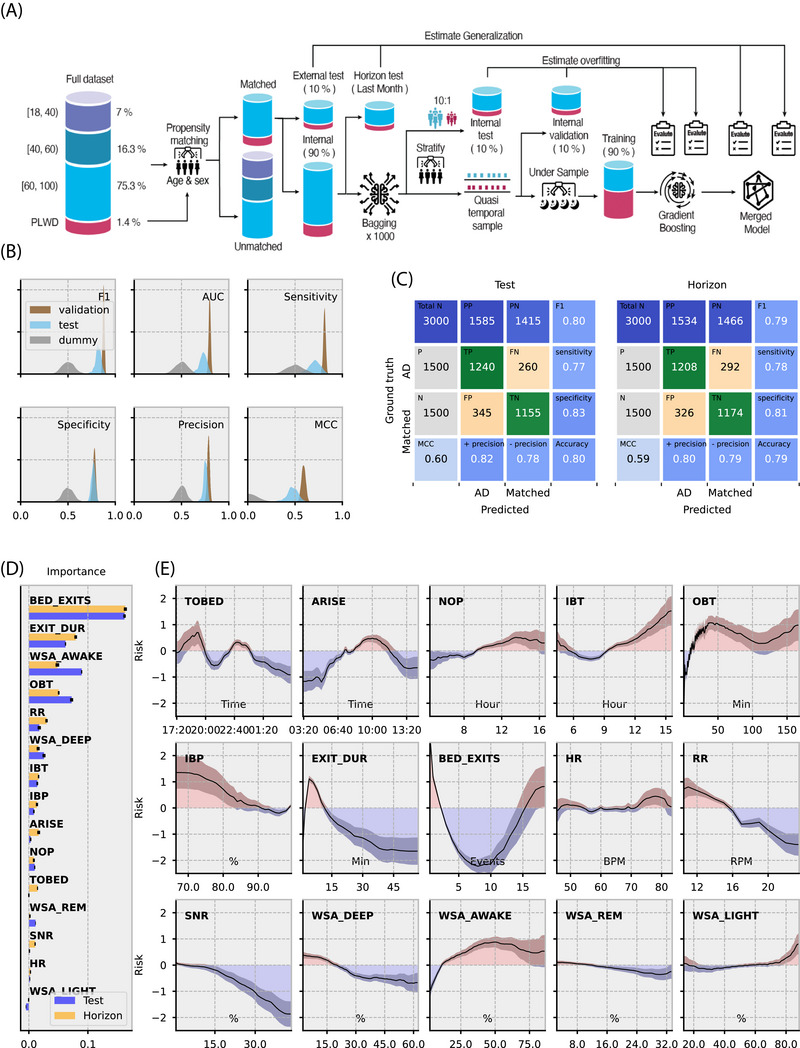
Machine learning model of Dementia Research Institute Sleep Index. A, Training pipeline: propensity matching is used to create a 1:25 case‐match dataset from the AD and the elderly population, generating a naïve dataset from unmatched observations. One thousand models are trained using a three‐step procedure to assess overfitting and generalization. First, 90% of the data are allocated to training and 10% to the test set. Next, quasi‐random sampling generates different temporal subsets for each individual. Models are trained on the training subset and tested on both the test and validation subsets. In parallel, null dummy models are trained on randomly shuffled labels, using the same algorithm for comparative analysis. B, Density ridge plots comparing density distributions for validation, training, and null across gold standard performance metrics including AUC and MCC. C, Confusion matrix of balanced samples from external and horizon test datasets applied over model night prediction; matched population (POP) and accuracy (ACC). D, Permutation post hoc importance test ordered feature contribution to classification performance on the two naïve test datasets. Bar *x* values represent the mean decrease in F1 score when feature values are shuffled. E, The Explainable Boosting Machine (EBM) computed the average influence exerted by each feature. The plots display the non‐linear association between risk and specific features, with areas corresponding to the general population highlighted in blue and dementia risk in red. Dark boundaries signify the confidence interval surrounding the risk for each metric score. Feature labels as in Table 1. AD, Alzheimer's disease; ARISE, time leaving bed; AUC, area under the curve; BED_EXITS, number of bed exits; EXIT_DUR, length of time out of bed; HR, heart rate; IBP, in‐bed proportion; IBT, in‐bed time; MCC, Matthews correlation coefficient; NOP, nocturnal occupancy period; OBT, out‐of‐bed time; RR, respiration rate; SNR, snoring; TO_BED, time to bed; WSA, Withings sleep analyzer; WSA_AWAKE, time awake estimate by Withings sleep analyzer; WSA_DEEP, deep sleep state estimate by Withings sleep analyzer; WSA_LIGHT, light sleep state estimate by Withings sleep analyzer; WSA_REM, rapid eye movement sleep estimate by Withings sleep analyzer.

To identify sleep patterns that differentiate individuals with AD from the general population, we developed the DRI‐SI‐AD. A machine‐learning framework based on an EBM was used (Figure 4A). The DRI‐SI‐AD captures key nocturnal sleep characteristics that distinguish AD from aging‐related changes in matched populations. Data from 83 individuals (40 males, age = 82.2 ± 7.7 years) with AD were compared to age‐ and sex‐matched observations from 790 individuals (400 males, age = 81.3 ± 7.5 years) from the WSA dataset. Individual nights were classified as originating from the AD or general population.

The EBM consistently outperformed the dummy model across all performance metrics and data subsets, suggesting stable performance across splits (see Table S4 in supporting information). On the internal test set, the EBM achieved an AUC of 0.82 (± 0.03), an F1 score of 0.73 (± 0.05), precision of 0.76 (± 0.02), sensitivity of 0.7 (± 0.7), and specificity of 0.77 (± 0.02; Figure 4B). The EBM's performance was slightly higher on the internal validation set, with an AUC of 0.88 (± 0.01), an F1 score of 0.80 (± 0.01), precision of 0.79 (± 0.01), sensitivity of 0.81 (± 0.02), and specificity of 0.78 (± 0.02). To further assess the final merged model, we first tested it on a balanced sample from external naïve test sets comprising entirely unseen individual nights (Figure 4C). This examination yielded an F1 score of 0.80, precision of 0.82 for the positive class and 0.78 for the negative class, sensitivity of 0.77, and specificity of 0.83. We further tested the model using another balanced sample from the horizon test, consisting of the excluded last 30 nights from all individuals used to train the merged models, achieved an F1 score of 0.79, precision of 0.8 for the positive class and 0.79 for the negative class, sensitivity of 0.78, and specificity of 0.81 (see Figure 4C).

Our machine‐learning approach allowed us to explore the relative contribution of different features to dementia classification (Figure 4D,E). Comparing AD to an age‐ and sex‐matched group from the general population, BED_EXITS and EXIT_DUR were the most important features for classification (Figure 4D). In general, a high risk of dementia was associated with early TO_BED, late ARISE, and prolonged IBT. AD tended to have many BED_EXITS that were relatively short (EXIT_DUR). Bed exits of relatively long duration were not associated with dementia. A relatively high time spent snoring was associated with a lower risk for dementia and a low respiratory rate was associated with a higher risk for dementia. The WSA_AWAKE was informative for dementia classification, with a high percentage of awake time related to an increased risk of AD.

### 3.6 Clinical relevance of DRI‐SI‐AD

The DRI‐SI‐AD may provide clinically relevant information for assessing AD and those at risk of dementia, which we demonstrate in three ways. First, we describe two clinical cases of dementia in detail in which the DRI‐SI‐AD nightly scores and clinical events are related. Second, we show the relationship between DRI‐SI‐AD and clinical measures of disease progression and dementia symptoms. Last, the model was applied to the population data (3,700,000 million nights across ≈ 13,500 individuals) to investigate the prevalence of DRI‐SI‐AD abnormalities in the general population.

#### 3.6.1 DRI‐SI‐AD reflects dementia progression and acute health events

**FIGURE 5:**
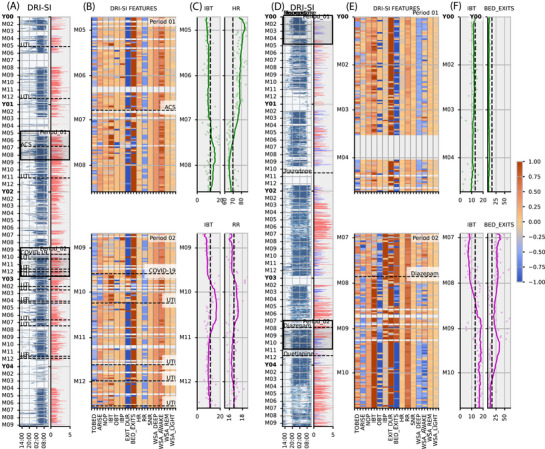
Data from two patients living with dementia. A, Data for an 83‐year‐old man with mixed AD and vascular dementia. First column night‐by‐night raster plots of bed occupancy (blue = in bed), coupled with Dementia Research Institute Sleep Index for Alzheimer's Disease (DRI‐SI‐AD) nightly absolute value (red indicates a high dementia risk, blue low risk). B, columns show DRI‐SI‐AD interpretable features for two comparative periods spanning 120 days in the middle of the feature plots for comparison (labels as in methods Table 1, upper and lower heatmaps). C, IBT, HR, and RR plotted for the 120 days showing scatter plots with overlayed rolling average (30‐day mean) line plots showing transient increases in IBT associated with acute coronary syndrome (ACS), COVID‐19 infection, and urinary tract infections. The dashed line shows the mean value for the features over the two comparative periods. D, Data for a 72‐year‐old woman with advanced Alzheimer's disease dementia and severe night‐time disturbance: layout (D‐F) as in (A‐C). F, IBT and BED_EXIT times show changes in night‐time behavior: stable in period 1 and disrupted in period 2 with extremely high number of BED_EXITS and low IBT, which improve after treatment with diazepam and quetiapine. AD, Alzheimer's disease; Arise, time getting up; AWAKE, awake period estimate; Bed, time going to bed; DEEP, deep sleep state estimate; Exit Duration, length of time spent out of the bed during nocturnal period; Exit Rate, rate of exits from bed during night; HR, heart rate IBT, in bed time; (BPM = beats per minute); LIGHT, light sleep estimate; Naps, length of time bed occupied outside nocturnal period; NOP, nocturnal occupancy period; OBT, out of bed time; REM, rapid eye movement estimate; RR, respiratory rate (RPM = respirations per minute); SNR, proportion of time spent snoring; TO_BED, time to bed; WSA, Withings sleep analyzer; WSA_AWAKE, time awake estimate by Withings sleep analyzer; WSA_DEEP, deep sleep state estimate by Withings sleep analyzer; WSA_LIGHT, light sleep state estimate by Withings sleep analyzer; WSA_REM, rapid eye movement sleep estimate by Withings sleep analyzer.

The DRI‐SI and underlying WSA features reflect the progression of dementia, the effects of medications, and co‐morbid medical events such as infections. We present two exemplar clinical cases tracked for > 2 years between 2019 and 2023, with nightly DRI‐SI‐AD scores and contributing features (Figure 5).

Case 1 (Figure 5A‐C) is an 83‐year‐old man with mixed AD and vascular dementia diagnosed in 2015 based on clinical history, imaging findings, and a neuropsychology profile. He has a complex past medical history including bipolar affective disorder, cervical laminectomy, aortic valve replacement with post‐operative lung hemorrhage requiring pneumonectomy, and neuropathic bladder after spinal surgery with indwelling catheter. He requires assistance with activities of daily living and has regular morning caregivers, which explains his very consistent ARISE time. Over the 4 years of monitoring, he has suffered from recurrent urinary tract infections (UTIs), which were accompanied by transient increases in IBT, and worsening of the DRI‐SI‐AD score. Similar sleep disruptions were observed in association with other acute illness including acute coronary syndrome in year 2 or COVID‐19 infection in year 3. Figure 5C first panel shows high HR in the period preceding an acute coronary event, with reductions after treatment. Figure 5C second panel shows RR increases after COVID infection and subsequent UTI, with a slightly delayed increase in the IBT.

Case 2 (Figure 5D‐F) is a 72‐year‐old woman with AD diagnosed in 2016 with typical amnestic presentation and neuroimaging appearances. She has a background of type 1 diabetes mellitus. Her disease progressed rapidly since the initial diagnosis with development of significant behavioral and psychological symptoms of dementia (BPSD), for which she was started on risperidone just before the trial start. After an initial period of stability in year 1 of monitoring (albeit with frequent BED_EXITS at night), her BPSD began to deteriorate again with frequent agitation and night‐time disturbance (e.g., trying to leave the house at night not knowing where she is) reflected in increased BED_EXITS and OBT, shorter IBT, and worsening DRI‐SI‐AD scores in years 2 to 4. Various pharmacological treatments have been trialed. Figure 5F first panel shows initially stable IBT and low numbers of bed exits. Figure 5F second panel shows lengthening of the time spent in bed after treatment with diazepam and eventual reduction in the number of BED_EXITS.

#### 3.6.2 DRI‐SI‐AD changes progressively with dementia progression and decline

**FIGURE 6:**
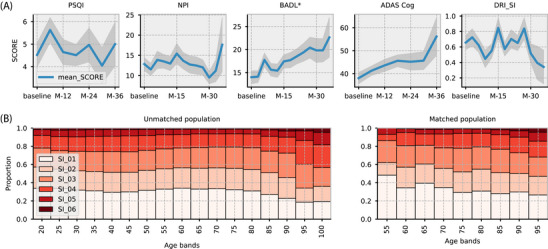
Association between Dementia Research Institute Sleep Index for Alzheimer's Disease (DRI‐SI‐AD) and Alzheimer's disease (AD) progression. A, Clinical scale score plots are shown over the acquisition period for AD (standard error in gray), for four disease progression scales: Pittsburgh Sleep Quality Index (PSQI), Neuropsychiatric Inventory (NPI), Bristol Activities of Daily Living (BADL), and Alzheimer Disease Assessment Scale Cognitive subscale (ADAS‐Cog). SCORE relates to the total score of a specific clinical scale (e.g., PSQI, BADL, etc.). * Indicates significant correlation between DRI‐SI‐AD and BADL scores. B, Stacked bar plots show the distribution of DRI‐SI‐AD scores across different age groups for both unmatched and matched datasets.

We next investigated the relationship between the DRI‐SI‐AD and AD progression. Standard disease progression clinical scales were assessed every 3 months (NPI and BADL) or every 6 months (PSQI and ADAS‐Cog) for 36 months, with a total of 1400 completed scales on 83 patients with AD. As expected, cognition declined as AD progressed (increasing ADAS‐Cog), and patients developed more functional problems (increasing BADL; Figure 6A). Neuropsychiatric symptoms and sleep disturbance measured by the NPI and PSQI fluctuated over the follow‐up period. Significant associations existed between DRI‐SI‐AD scores measured 90 days before each assessment and functional assessments. Using mixed effects linear models, we estimated the association of the mean DRI‐SI‐AD over that period while controlling for age and sex. DRI‐SI‐AD was significantly associated with BADL scores (coef. = 0.835 ± 0.316, *Z* = 2.613, *p* = 0.009). There was no significant association between PSQI, ADAS‐Cog, or NPI and DRI‐SI‐AD.

Finally, we applied the DRI‐SI‐AD to both the matched (*n* = 792, nights = 360,635) and unmatched (*n* = 12,796, nights = 3,425,717) unfiltered population data to label individuals’ WSA recordings based on similarity to our AD recordings. DRI‐SI‐AD scores were classified night‐by‐night into the categories (1–6), with 6 being the most like our AD group and 1 being the least similar (Figure 6B). From ≈ 65 years of age, the proportion of night sleep categorized as 3 to 6 steadily increased with a proportionate decrease in categories 1 and 2. Similar results were seen in the unmatched naïve dataset in which data had not been used to generate the DRI‐SI‐AD.

## 4 DISCUSSION

Sleep disturbances in dementia are under‐characterized. Using affordable, non‐invasive under‐mattress sensors and machine learning, we continuously monitored home sleep and physiology. Compared to normal aging, PLWD show earlier, longer bedtimes and frequent awakenings, yet patterns differ widely between individuals and shift over years. These signatures, detectable even in the general population, are summarized by our DRI‐SI‐AD, which mirrors acute health changes and could potentially inform clinical care.

Sleep disturbance rises with age and is an early marker and potential driver of neurodegeneration.^5^, ^7^, ^40^, ^41^, ^42^, ^43^ Manifestations include abnormal timing, inconsistent duration, frequent interruptions, and nocturnal wandering. Common disorders—sleep apnea, REM sleep behavior disorder—and broader changes in sleep structure are frequently observed. Disrupted architecture is a hallmark of many neurodegenerative diseases, ^41^, ^43^ occurring in 65% of AD cases, and 90% of Parkinson's disease dementia.^43^ Established AD sleep disturbance is a known predictor of nursing home admission.^5^ In healthy older adults it raises the likelihood of developing AD by 50%.^7^ Sleep problems are also an early feature of Parkinson's disease (PD), often preceding diagnosis,^42^ with evidence that sleep disturbance plays a pathogenic role in PD.^40^

Collecting objective sleep information at home has been challenging because gold standard, rigorous sleep‐laboratory studies are limited and short, and wearables still have limited utility in patients with cognitive impairment. Home monitoring with actigraphy and wearables can be burdensome for such patients, and these recordings typically capture only very brief periods.^44^, ^45^ The WSA, a non‐contact under‐mattress device, enables long‐term, unobtrusive data collection in real‐world home environments. Unlike smartwatches, the contactless sensor passively collects sleep data, accurately capturing in‐bed timing, interruptions, and cardio‐respiratory signals in older adults and those with dementia.^22^

Our approach complements standard self‐ or informant‐reported measures, as shown in relation to the PSQI (see Figure S3). It adds features that are difficult to obtain via self‐report, such as detailed bed occupancy, minute‐by‐minute pulse and respiratory rate, and sleep stage estimates. This is especially valuable for individuals with cognitive impairment, who often struggle to report night‐time behavior accurately.

Specifically, our findings confirm that aging is linked to earlier sleep onset, offset, and more frequent interruptions. For example, rise times in AD are later than those aged 60 to 100 and time spent in bed is longer. A prominent aspect of sleep in AD is the combination of prolonged nocturnal bedtimes with frequent disruptions, significantly impacting sleep continuity.

Long self‐reported sleep durations and disrupted sleep continuity are established risk factors for cognitive decline, notably in AD.^46^ Our approach enables these risk factors to be assessed objectively and continuously. They also emphasize the importance of the frequency and duration of bed exits, features not quantified in previous studies. Sleep apnea is also a risk factor for dementia and is highly prevalent in AD. Our data show that reduced respiratory snoring rates were observed more often in AD. One possibility is that reductions in inspiratory effort and increased central sleep apnea may underlie the association between reduced respiration rate and snoring time in dementia.^47^ In addition, alterations in awake periods, deep and REM sleep were all seen in AD compared to an age‐matched population. Reduced deep and REM sleep were observed, along with an increase in awake periods. These findings agree with several PSG studies, although they did not emerge as important features in our machine‐learning model. This may be related to the poor accuracy of the device in detecting sleep structure, which may also explain the observed age‐related increase in deep sleep, which is at odds with many PSG studies in aging.^18^

A key question is whether sleep disturbances in AD reflect aging or neurodegeneration. Our data suggest both contribute and we have used propensity matching used to separate their effects. Whole‐group comparisons (Figure 2) show shared changes like earlier bedtimes, although these are more pronounced in AD. Other features, such as later arise times and reduced snoring, appear AD specific. Moreover, explainable machine learning suggests that night‐by‐night sleep features may help differentiate AD from matched controls, with sleep continuity metrics (e.g., bed exits, exit duration) being most discriminatory. After propensity matching, between‐group differences were often modest as there are large age effects that are controlled for in this analysis and significant variability across the AD group. We further explored this variability using unsupervised clustering (Figure 3) and explainable supervised modeling to identify sleep features that showed disease‐related variation after controlling for age effects (Figure 4).

We developed the DRI‐SI‐AD using explainable machine learning to integrate night‐by‐night data into a single digital biomarker. The DRI‐SI‐AD appears to capture AD‐related sleep abnormalities that can be tracked over months or years. It complements standard self‐ or informant‐reported measures, as shown with the PSQI, and includes features not accessible through self‐report, such as bed occupancy, minute‐level pulse and respiratory data, and sleep stages approximation. This makes it especially valuable for individuals with cognitive impairment who struggle to report night‐time behavior.

The DRI‐SI‐AD shows reasonable AUCs for classifying AD patients using night‐by‐night information. However, the value of the continuous sleep monitoring and analytics we present is not designed to compete with or replace simple clinical assessments such as the Mini‐Mental State Examination. Rather, as a continuous digital biomarker, it can be used for objective sleep monitoring over long periods. In addition, it is possible that this type of continuous monitoring might complement traditional screening tools by identifying early signs of deterioration or by providing real‐time feedback on the effectiveness of interventions.

Increasing DRI‐SI‐AD scores were associated with behavioral disturbances measured by the BADL but no other clinical measures. As the BADL quantifies functional ability in everyday life this suggests that our index may be capturing aspects of sleep linked to functional decline. In practical terms, this indicates that the DRI‐SI‐AD could serve as an objective tool for monitoring sleep disturbance in AD that is relevant to daytime function and may provide a sensitive way to assess the impact of interventions aimed at improving sleep and night‐time behavior.

The DRI‐SI‐AD has been developed as a modular, data‐driven pipeline. It is, in principle, sensor agnostic as the model can be generated using similar features derived from a different device. The computed features, for example, time in bed and so on, are designed to be interpretable as independent observations and can be used separately from the DRI‐SI. The derived features can be calculated automatically on data streamed from the device, which can then be shared through a companion app.

The sleep disturbances we commonly observed in AD may be indicators of dementia risk in those with preclinical AD. The presence of neurodegenerative pathology alone is insufficient to identify who will develop AD. For example, many people with high brain amyloid levels remain symptom free. Functional measures of the behavioral impact of neurodegenerative pathology may provide valuable information about an individual's proximity to dementia. We have shown that the DRI‐SI‐AD appears sensitive to dementia like sleep disturbances in the general population and future work could test the hypothesis that sleep disturbances may predict the presence of AD pathology and/or the probability of transition to dementia in individuals with AD pathology but no overt signs of cognitive impairment or dementia.

The study has several limitations. We have previously shown that the WSA estimation of sleep stages does not accurately reflect gold standard estimates. Hence, more research needs to be performed to understand the utility of sleep stages estimated in this way. In addition, we had relatively sparse clinical assessment, which limited our ability to understand night‐by‐night variability in clinical measures in relation to DRI‐SI‐AD fluctuations. These fluctuations are likely relevant to variability, but this will need to be studied with more granular clinical measures.

In our full dataset, the number of general population cases greatly exceeds AD cases by a ratio of ≈ 164:1. This significant imbalance can bias the machine learning model, causing it to overly focus on patterns typical in the more substantial (majority) group and neglect the unique sleep features explicitly related to AD (the minority class). To overcome this, we trained our EBM on a balanced subset, using a 1:10 matching ratio—in which each AD case is paired with 10 general population cases. This approach maintains a representative yet manageable sample that reflects the true prevalence of AD, ensuring the model can effectively learn and highlight sleep patterns uniquely associated with AD, without being dominated by the majority group's data.

A further limitation is the modest number of participants with AD (*N* = 83), which is a challenge given the heterogeneity of sleep disturbances produced by the disease. Our approach provides extensive individual assessment, but a comprehensive characterization of the effects of AD on sleep and night‐time behavior will require a larger AD group at a range of disease stages. Our approach is potentially saleable to much larger patient groups, so this is an achievable goal for future work.

In summary, our work demonstrates ecological validity in that information of relevance to dementia can be recorded over long periods from the homes of individuals with and without dementia in a way that has previously never been done. The sensor is low cost and well suited for prolonged monitoring. Our findings suggest that implementation of this contactless technology at scale in combination with platforms to visualize and analyze the data holds promise for the longitudinal assessments of sleep and night‐time behavior in PLWD.

## CONFLICT OF INTEREST STATEMENT

The authors declare no conflicts of interest relevant to this work. Author disclosures are available in the supporting information.

## CONSENT STATEMENT

All participants provided written informed consent to participate in this study. For participants unable to provide consent due to cognitive impairment, consent was obtained from their legal representatives or caregivers in accordance with the UK Mental Capacity Act 2005.

## Supporting information



Supporting Information

Supporting Information

Supporting Information

Supporting Information

Supporting Information

## References

[1] Benca R , Teodorescu M . Sleep physiology and disorders in aging and dementia. Handb Clin Neurol. 2019;167:477‐493. 31753150 10.1016/B978-0-12-804766-8.00026-1

[2] Koren T , Fisher E , Webster L , Livingston G , Rapaport P . Prevalence of sleep disturbances in people with dementia living in the community: a systematic review and meta‐analysis. Ageing Res Rev. 2023;83:101782. 36356799 10.1016/j.arr.2022.101782

[3] Zhang Y , Ren R , Yang L , et al. Sleep in Alzheimer's disease: a systematic review and meta‐analysis of polysomnographic findings. Translational psychiatry. 2022;12:136. 35365609 10.1038/s41398-022-01897-y PMC8976015

[4] Matsumoto S , Tsunematsu T . Association between sleep, Alzheimer's, and Parkinson's disease. Biology. 2021;10:1127. 34827122 10.3390/biology10111127 PMC8614785

[5] Bianchetti A , Scuratti A , Zanetti O , et al. Predictors of mortality and institutionalization in Alzheimer disease patients 1 year after discharge from an Alzheimer dementia unit. Dementia. 1995;6:108‐112. 7606278 10.1159/000106930

[6] Hope T , Keene J , Gedling K , Fairburn C , Jacoby R . Predictors of institutionalization for people with dementia living at home with a carer. International journal of geriatric psychiatry. 1998;13:682‐690. 9818303 10.1002/(sici)1099-1166(1998100)13:10<682::aid-gps847>3.0.co;2-y

[7] Shi L , Chen S , Ma M , et al. Sleep disturbances increase the risk of dementia: a systematic review and meta‐analysis. Sleep Med Rev. 2018;40:4‐16. 28890168 10.1016/j.smrv.2017.06.010

[8] Guarnieri B , Adorni F , Musicco M , et al. Prevalence of sleep disturbances in mild cognitive impairment and dementing disorders: a multicenter Italian clinical cross‐sectional study on 431 patients. Dement Geriatr Cogn Disord. 2012;33:50‐58. 22415141 10.1159/000335363 PMC3696366

[9] Fenton L , Isenberg AL , Aslanyan V , et al. Variability in objective sleep is associated with Alzheimer's pathology and cognition. Brain Communications. 2023;5:fcad031. 36895954 10.1093/braincomms/fcad031 PMC9989141

[10] Wennberg A , Wu M , Rosenberg P , Spira A . Sleep Disturbance, Cognitive Decline, and Dementia: a Review. Semin Neurol. 2017;37:395‐406. 28837986 10.1055/s-0037-1604351 PMC5910033

[11] Yaffe K , Falvey C , Hoang T . Connections between sleep and cognition in older adults. The Lancet Neurology. 2014;13:1017‐1028. 25231524 10.1016/S1474-4422(14)70172-3

[12] Li P , Gao L , Gaba A , et al. Circadian disturbances in Alzheimer's disease progression: a prospective observational cohort study of community‐based older adults. Lancet Healthy Longev. 2020;1:e96‐e105. 34179863 10.1016/s2666-7568(20)30015-5 PMC8232345

[13] Todd W . Potential Pathways for Circadian Dysfunction and Sundowning‐Related Behavioral Aggression in Alzheimer's Disease and Related Dementias. Front Neurosci. 2020;14:910. 33013301 10.3389/fnins.2020.00910 PMC7494756

[14] D'Rozario AL , Chapman JL , Phillips CL , et al. Objective measurement of sleep in mild cognitive impairment: a systematic review and meta‐analysis. Sleep Medicine Reviews. 2020;52:101308. 32302775 10.1016/j.smrv.2020.101308

[15] Balouch S , Dijk DA , Rusted J , Skene SS , Tabet N , Dijk D . Night‐to‐night variation in sleep associates with day‐to‐day variation in vigilance, cognition, memory, and behavioral problems in Alzheimer's disease. Alzheimers Dement (Amst). 2022;14:e12303. 35603140 10.1002/dad2.12303 PMC9109375

[16] Ferini‐Strambi L , Liguori C , Lucey BP , et al. Role of sleep in neurodegeneration: the consensus report of the 5th Think Tank World Sleep Forum. Neurological sciences. 2024;45:749‐767. 38087143 10.1007/s10072-023-07232-7

[17] Van Erum J , Van Dam D , De Deyn P . Sleep and Alzheimer's disease: a pivotal role for the suprachiasmatic nucleus. Sleep Med Rev. 2018;40:17‐27. 29102282 10.1016/j.smrv.2017.07.005

[18] Romanella S , Roe D , Tatti E , et al. The sleep side of aging and Alzheimer's disease. Sleep medicine. 2021;77:209‐225. 32912799 10.1016/j.sleep.2020.05.029 PMC8364256

[19] Moore K , O'Shea E , Kenny L , et al. Older Adults' Experiences With Using Wearable Devices: qualitative Systematic Review and Meta‐synthesis. JMIR Mhealth Uhealth. 2021;9:e23832. 34081020 10.2196/23832 PMC8212622

[20] Bloom HG , Ahmed I , Alessi CA , et al. Evidence‐based recommendations for the assessment and management of sleep disorders in older persons. J Am Geriatr Soc. 2009;57:761‐789. 19484833 10.1111/j.1532-5415.2009.02220.x PMC2748127

[21] Kononova A , Li L , Kamp K , et al. The Use of Wearable Activity Trackers Among Older Adults: focus Group Study of Tracker Perceptions, Motivators, and Barriers in the Maintenance Stage of Behavior Change. JMIR Mhealth Uhealth. 2019;7:e9832. 30950807 10.2196/mhealth.9832 PMC6473213

[22] Tal A , Shinar Z , Shaki D , Codish S , Goldbart A . Validation of Contact‐Free Sleep Monitoring Device with Comparison to Polysomnography. J Clin Sleep Med. 2017;13:517‐522. 27998378 10.5664/jcsm.6514 PMC5337599

[23] Ravindran KKG , della Monica C , Atzori G , et al. Contactless and longitudinal monitoring of nocturnal sleep and daytime naps in older men and women: a digital health technology evaluation study. Sleep. 2023;46:zsad194. 37471049 10.1093/sleep/zsad194 PMC10566241

[24] Edouard P , Campo D , Bartet P , et al. Validation of the Withings Sleep Analyzer, an under‐the‐mattress device for the detection of moderate‐severe sleep apnea syndrome. J Clin Sleep Med. 2021;17:1217‐1227. 33590821 10.5664/jcsm.9168 PMC8314651

[25] Lee XK , Chee NI , Ong JL , et al. Validation of a Consumer Sleep Wearable Device With Actigraphy and Polysomnography in Adolescents Across Sleep Opportunity Manipulations. J Clin Sleep Med. 2019;15:1337‐1346. 31538605 10.5664/jcsm.7932 PMC6760396

[26] G Ravindran KK , della Monica C , Atzori G , et al. Three Contactless Sleep Technologies Compared With Actigraphy and Polysomnography in a Heterogeneous Group of Older Men and Women in a Model of Mild Sleep Disturbance: sleep Laboratory Study. JMIR mHealth and uHealth. 2023;11:e46338. 37878360 10.2196/46338 PMC10632916

[27] McKhann G , Drachman D , Folstein M , Katzman R , Price D , Stadlan EM . Clinical diagnosis of Alzheimer's disease: report of the NINCDS‐ADRDA Work Group under the auspices of Department of Health and Human Services Task Force on Alzheimer's Disease. Neurology. 1984;34:939‐944. 6610841 10.1212/wnl.34.7.939

[28] Bucks R , Ashworth D , Wilcock G , Siegfried K . Assessment of activities of daily living in dementia: development of the Bristol Activities of Daily Living Scale. Age Ageing. 1996;25:113‐120. 8670538 10.1093/ageing/25.2.113

[29] RT P , Taylor R , Minor BL , et al. The REDCap consortium: building an international community of software partners. J Biomed Inform. 2019;95:103208. 31078660 10.1016/j.jbi.2019.103208 PMC7254481

[30] della Monica C , Ravindran KKG , Atzori G , et al. A protocol for evaluating digital technology for monitoring sleep and circadian rhythms in older people and people living with dementia in the community. Clocks & Sleep. 2024;6:129‐155. 38534798 10.3390/clockssleep6010010 PMC10968838

[31] Ravindran K , et al. Reliable Contactless Monitoring of Heart Rate, Breathing Rate and Breathing Disturbance During Sleep in Aging: A Digital Health Technology Evaluation Study. (2023). 10.2196/53643 PMC11387924 39190477

[32] Rosenbaum P , Rubin D . The central role of the propensity score in observational studies for causal effects. Biometrika. 1983;70:41‐55.

[33] Zimmerman D . A note on preliminary tests of equality of variances. Br J Math Stat Psychol. 2004;57:173‐181. 15171807 10.1348/000711004849222

[34] Kailath T . The divergence and Bhattacharyya distance measures in signal selection. IEEE transactions on communication technology. 1967;15:52‐60.

[35] Rao C . A review of canonical coordinates and an alternative to correspondence analysis using Hellinger distance. Qüestiió: Quaderns d'estadística i investigació operativa. 1995.

[36] Nori H , Jenkins S , Koch P , Caruana R , Interpretml: A unified framework for machine learning interpretability. arXiv preprint arXiv:1909.09223 (2019).

[37] Saeb S , Lonini L , Jayaraman A , Mohr D , Kording K . The need to approximate the use‐case in clinical machine learning. Gigascience. 2017;6:1‐9. 10.1093/gigascience/gix019 PMC5441397 28327985

[38] Raschka S , Model evaluation, model selection, and algorithm selection in machine learning. arXiv preprint arXiv:1811.12808 (2018).

[39] He H , Garcia E . Learning from imbalanced data. IEEE Transactions on knowledge and data engineering. 2009;21:1263‐1284.

[40] Bohnen N , Hu M . Sleep Disturbance as Potential Risk and Progression Factor for Parkinson's Disease. J Parkinsons Dis. 2019;9:603‐614. 31227656 10.3233/JPD-191627 PMC6700634

[41] Diekelmann S , Born J . The memory function of sleep. Nat Rev Neurosci. 2010;11:114‐126. 20046194 10.1038/nrn2762

[42] Iranzo A , Fernández‐Arcos A , Tolosa E , et al. Neurodegenerative disorder risk in idiopathic REM sleep behavior disorder: study in 174 patients. PLoS One. 2014;9:e89741. 24587002 10.1371/journal.pone.0089741 PMC3935943

[43] Krause AJ , Simon EB , Mander BA , et al. The sleep‐deprived human brain. Nat Rev Neurosci. 2017;18:404‐418. 28515433 10.1038/nrn.2017.55 PMC6143346

[44] Thorpy M . International classification of sleep disorders. Sleep disorders medicine: Basic science, technical considerations and clinical aspects. 2017:475‐484.

[45] Jiménez‐Jiménez FJ , Alonso‐Navarro H , García‐Martín E , Agúndez JAG . Current Treatment Options for REM Sleep Behaviour Disorder. J Pers Med. 2021;11:1204. 34834556 10.3390/jpm11111204 PMC8624088

[46] Winer JR , Lok R , Weed L , et al. Impaired 24‐h activity patterns are associated with an increased risk of Alzheimer's disease, Parkinson's disease, and cognitive decline. Alzheimers Res Ther. 2024;16:35. 38355598 10.1186/s13195-024-01411-0 PMC10865579

[47] Ercolano E , Bencivenga L , Palaia ME , et al. Intricate relationship between obstructive sleep apnea and dementia in older adults. Geroscience. 2024;46:99‐111. 37814196 10.1007/s11357-023-00958-4 PMC10828345

