# Supplementary material for: Contactless longitudinal monitoring in the home characterizes aging and Alzheimer's disease–related night‐time behavior and physiology
Source: Alzheimers Dement. 2025 Oct 25;21(10):e70758. doi: 10.1002/alz.70758 (PMC12552897; doi:10.1002/alz.70758)
Supplement: Supplementary file 1 — Supporting Information [file ALZ-21-e70758-s001.pdf]

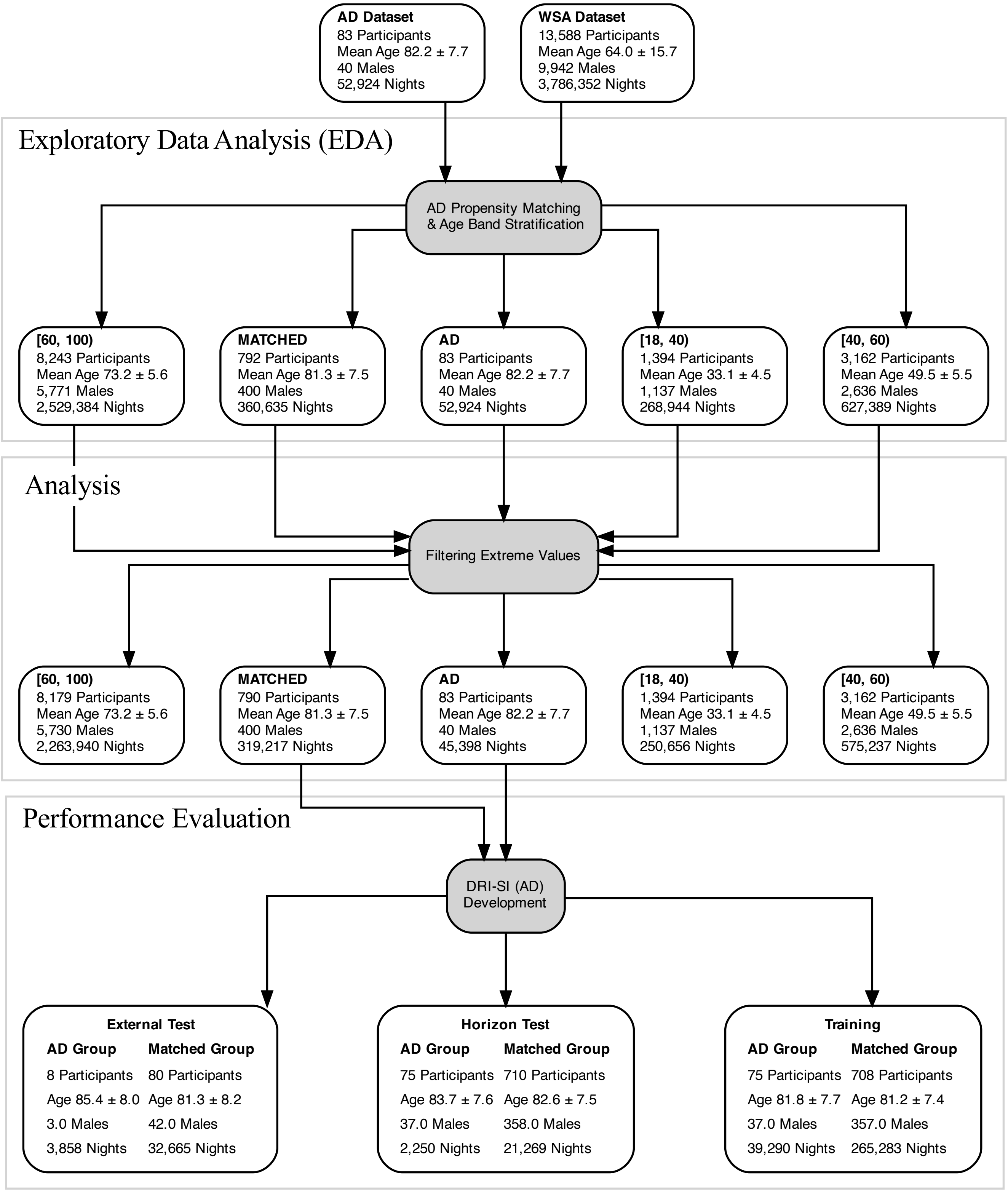

Supplementary Figure 1:
consort diagram communicating the different sensor input datasets used in each stage of the study.
