# Supplementary material for: Contactless longitudinal monitoring in the home characterizes aging and Alzheimer's disease–related night‐time behavior and physiology
Source: Alzheimers Dement. 2025 Oct 25;21(10):e70758. doi: 10.1002/alz.70758 (PMC12552897; doi:10.1002/alz.70758)
Supplement: Supplementary file 2 — Supporting Information [file ALZ-21-e70758-s005.pdf]

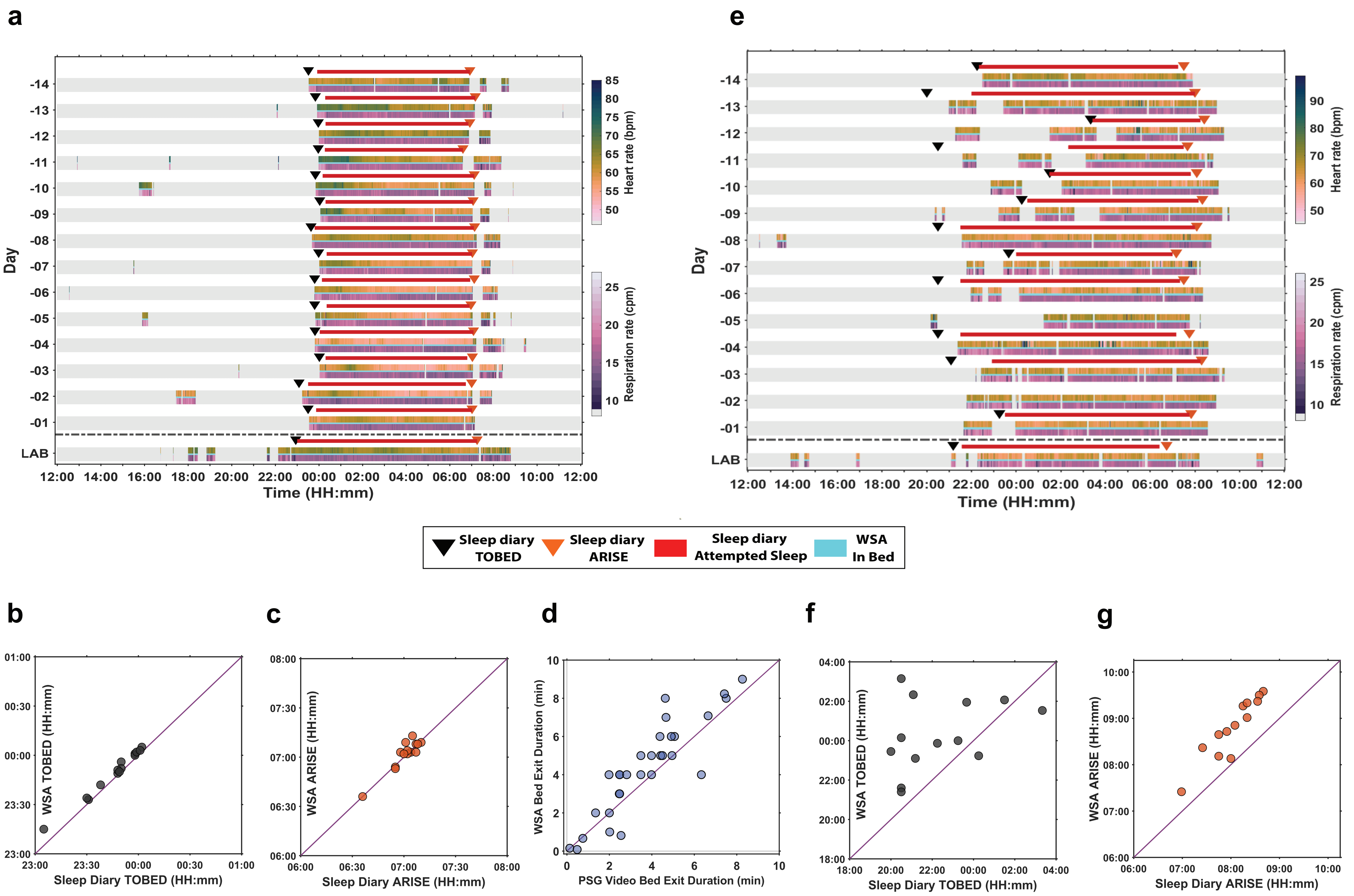

Supplementary Figure 2

Fourteen days of continuous home recording followed by one overnight recording in a sleep laboratory using the WSA in a (a) healthy older adult and (e) a person living with Alzheimer's. Variables displayed: WSA- bed-occupancy period, WSA-heart rate (beats per minute) and WSA-respiration rate (cycles per minute) along with the consensus sleep diary reported TOBED and ARISE time and Attempted to Sleep Period; (b and f) TOBED and (c and g) ARISE times recorded from the WSA plotted against the corresponding consensus sleep diary entries for the data depicted in (a and e). Please note the close association between the self-reported and WSA detected TOBED and ARISE time in healthy older adult and weaker association between the self-reported and objectively assessed times in the AD; (d) Duration of Bed Exits (minutes) from the WSA plotted against polysomnography video assessed Duration of Bed Exits over the lights-off period during overnight laboratory recordings in (N=30 bed exits observed in 35 Older adults).
