# Supplementary material for: Contactless longitudinal monitoring in the home characterizes aging and Alzheimer's disease–related night‐time behavior and physiology
Source: Alzheimers Dement. 2025 Oct 25;21(10):e70758. doi: 10.1002/alz.70758 (PMC12552897; doi:10.1002/alz.70758)
Supplement: Supplementary file 3 — Supporting Information [file ALZ-21-e70758-s003.pdf]

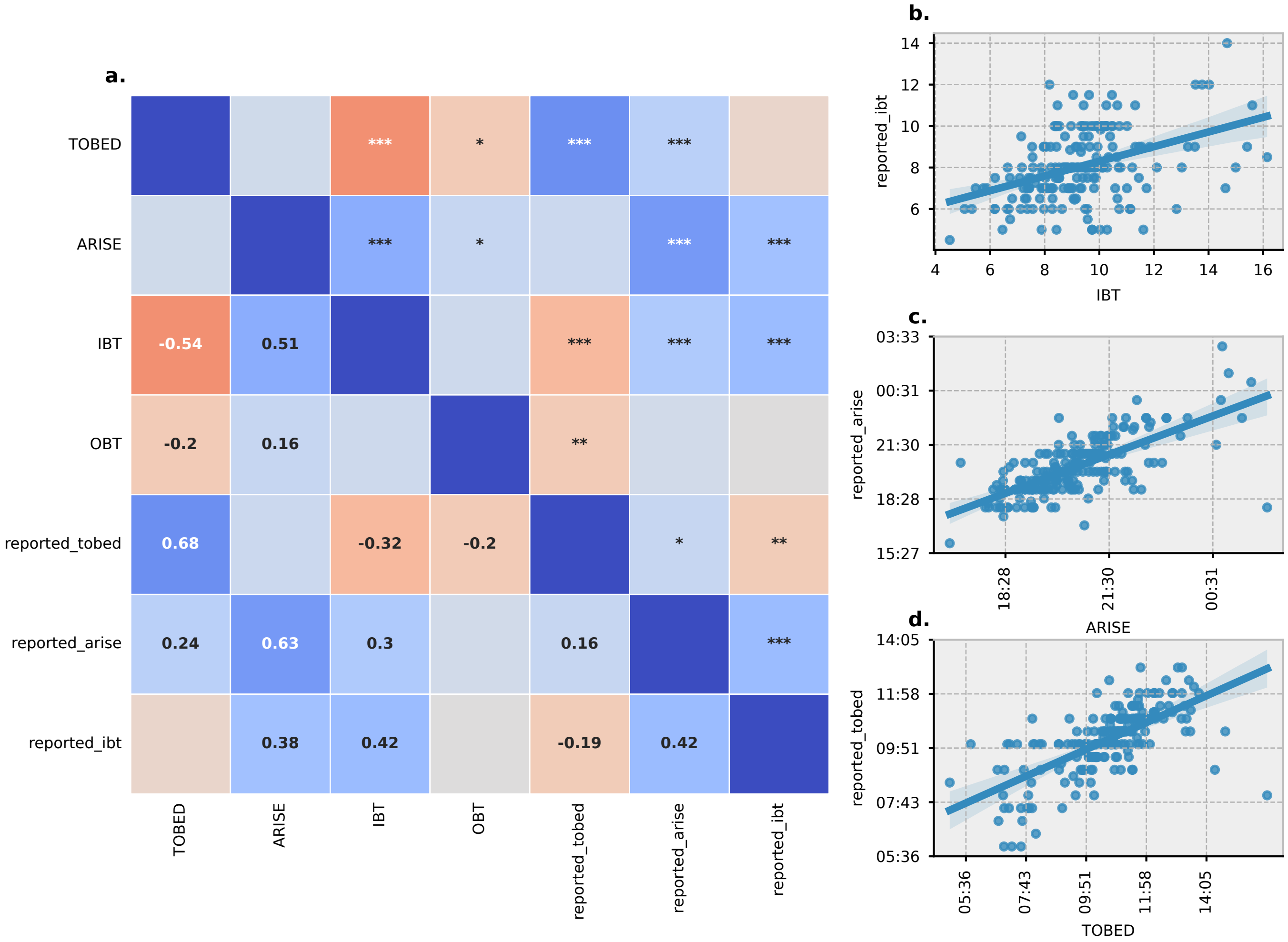

Supplementary Figure 3

a. Spearman correlation matrix of objective WSA measures from 90 days preceding subjective reporting using the PSQI scale. b-d. Regression scatter plots comparing subjective reported PSQI measures with WSA objective measurements of in bed time (IBT), time to be (TOBED) and time out of bed (ARISE).
