# Supplementary material for: Contactless longitudinal monitoring in the home characterizes aging and Alzheimer's disease–related night‐time behavior and physiology
Source: Alzheimers Dement. 2025 Oct 25;21(10):e70758. doi: 10.1002/alz.70758 (PMC12552897; doi:10.1002/alz.70758)
Supplement: Supplementary file 4 — Supporting Information [file ALZ-21-e70758-s002.docx]

**Supplementary Table 1 – WSA demographics and feature aggregates by age band**

|  | **Age Groups** | | | | |
| --- | --- | --- | --- | --- | --- |
|  | **AD** | **MATCHED** | **[60, 100)** | **[40, 60)** | **[18, 40)** |
| **Individuals** | 83 | 792 | 8243 | 3162 | 1394 |
| **Nights** | 52,924 | 360,635 | 2,529,384 | 627,389 | 268,944 |
| **Age (years)** | 83.2 ± 7.8 | 82.6 ± 6.8 | 73.2 ± 4.6 | 49.9 ± 5.5 | 33.6 ± 4.4 |
| **Males** | 40 | 400 | 5,771 | 2,635 | 1,136 |
| **Females** | 43 | 392 | 2,471 | 526 | 257 |
| **TOBED** | 22:47 ± 2.8 hours | 22:46 ± 2.1 hours | 23:05 ± 1.9 hours | 23:38 ± 1.7 hours | 23:59 ± 1.9 hours |
| **ARISE** | 08:29 ± 2.4 hours | 08:00 ± 3.1 hours | 07:52 ± 2.5 hours | 08:14 ± 2.1 hours | 08:50 ± 2.1 hours |
| **NOP** | 9.8 ± 3.3 hours | 9.2 ± 3.3 hours | 8.7 ± 2.6 hours | 8.6 ± 2.1 hours | 8.9 ± 1.9 hours |
| **IBT** | 9.3 ± 3.1 hours | 8.7 ± 2.8 hours | 8.4 ± 2.2 hours | 8.4 ± 1.8 hours | 8.7 ± 1.6 hours |
| **OBT** | 33 ± 62.9 min | 29 ± 72.2 min | 22 ± 66.4 min | 11 ± 43.5 min | 11 ± 41.0 min |
| **BED_EXITS** | 3.5 ± 5.5 events | 3.6 ± 2.9 events | 3.1 ± 2.5 events | 2.3 ± 2.2 events | 2.3 ± 2.1 events |
| **IBP** | 94.8% ± 9.2 | 95.4% ± 8.1 | 96.3% ± 7.1 | 98.0% ± 4.0 | 98.1% ± 4.0 |
| **EXIT_DUR** | 9.6 ± 23.9 min | 8.7 ± 26.5 min | 7.4 ± 24.8 min | 4.6 ± 19.2 min | 4.3 ± 17.3 min |
| **HR (**BPM) | 60.7 ± 7.6 BPM | 60.5 ± 7.6 BPM | 60.7 ± 8.1 BPM | 62.1 ± 8.3 BPM | 60.8 ± 7.9 BPM |
| **RR (**RPM) | 15.0 ± 2.2 RPM | 15.8 ± 2.5 RPM | 15.4 ± 2.3 RPM | 15.2 ± 2.2 RPM | 15.4 ± 2.1 RPM |
| **SNR** | 3.2% ± 5.9 | 5.2% ± 9.5 | 8.0% ± 14.9 | 8.8% ± 13.0 | 3.9% ± 8.0 |
| **WSA_DEEP** | 15.5% ± 13.7 | 22.1% ± 15.2 | 23.4% ± 13.8 | 19.9% ± 10.3 | 18.4% ± 9.1 |
| **WSA_AWAKE** | 22.3% ± 18.3 | 16.0% ± 13.9 | 13.8% ± 11.2 | 11.4% ± 8.1 | 12.5% ± 7.9 |
| **WSA_REM** | 9.0% ± 8.2 | 10.7% ± 8.5 | 12.2% ± 8.6 | 16.8% ± 8.7 | 17.7% ± 8.7 |
| **WSA_LIGHT** | 53.2% ± 17.3 | 51.1% ± 16.2 | 50.6% ± 15.1 | 51.9% ± 13.1 | 51.3% ± 12.6 |

**Supplementary Table 1: WSA Demographics and Feature Aggregates by Age Band**
This table presents demographic data and aggregated sleep feature statistics across different age groups, including individuals with Alzheimer's Disease (AD), matched controls, and the general population. Metrics such as time to bed (TOBED), wake-up time (ARISE), in-bed time (IBT), out-of-bed time (OBT), and heart rate (HR) are included to compare nocturnal behaviours. This table highlights the variation in sleep and physiological patterns with ageing and dementia.

**Supplementary Table 2 – AD Sleep Pattern Phenotypes**

| **clusters** | **Name** | **Obs** | **Age** | **IBT** | **OBT** | **BED_EXITS** | **EXIT_DUR** | **TOBED** | **ARISE** |
| --- | --- | --- | --- | --- | --- | --- | --- | --- | --- |
| **cluster_1** | **Very Early, Fragmented Bed Occupancy** | 6,618 | 86.5 ± 6.6 | 11.8 ± 1.9 | 35.8 ± 39.5 | 4.0 ± 3.9 | 8.4 ± 9.9 | 19:30 ± 00:56 | 07:50 ± 01:49 |
| **cluster_2** | **Early to Bed and Rise** | 11,558 | 82.5 ± 7.3 | 8.5 ± 1.1 | 15.3 ± 21.6 | 2.4 ± 2.5 | 5.5 ± 6.0 | 22:24 ± 00:38 | 07:10 ± 00:53 |
| **cluster_3** | **Late Rising, Disrupted Bed Occupancy** | 2,305 | 81.9 ± 6.5 | 12.4 ± 1.4 | 28.2 ± 24.2 | 4.1 ± 3.1 | 7.6 ± 7.0 | 22:40 ± 01:04 | 11:30 ± 01:14 |
| **cluster_4** | **Prolonged Bed Occupancy** | 13,308 | 82.6 ± 7.9 | 9.7 ± 0.9 | 18.8 ± 21.8 | 2.9 ± 2.8 | 6.2 ± 6.1 | 23:00 ± 00:42 | 09:03 ± 00:34 |
| **cluster_5** | **Normative Bed Occupancy** | 5,176 | 82.0 ± 8.7 | 6.8 ± 1.0 | 14.2 ± 19.9 | 2.2 ± 3.0 | 6.0 ± 6.7 | 00:05 ± 00:44 | 07:04 ± 00:36 |
| **cluster_6** | **Delayed Bed Timing** | 6,433 | 82.9 ± 8.0 | 8.2 ± 1.7 | 16.0 ± 23.0 | 2.0 ± 2.4 | 6.5 ± 7.9 | 01:29 ± 01:02 | 09:58 ± 01:34 |

**Supplementary Table 2: AD Sleep Pattern Phenotypes**
This table categorizes distinct sleep pattern phenotypes observed in individuals with Alzheimer's Disease (AD) using hierarchical clustering. Metrics such as in-bed time (IBT), bed exits, and exit durations provide insights into unique behavioural characteristics across six clusters. The table illustrates how sleep timing, duration, and interruptions differ within the AD population.

**Supplementary Table 3 – Performance values for occupancy multi-class pattern classification model.**

| **PID** | **nights** | **precision** | **f1** | **sensitivity** | **accuracy** | **PID** | **nights** | **precision** | **f1** | **sensitivity** | **accuracy** |
| --- | --- | --- | --- | --- | --- | --- | --- | --- | --- | --- | --- |
| **1** | 166 | 0.99 | 0.99 | 0.99 | 0.99 | **44** | 649 | 0.94 | 0.94 | 0.94 | 0.94 |
| **2** | 1,022 | 0.83 | 0.80 | 0.80 | 0.80 | **45** | 126 | 0.99 | 0.99 | 0.99 | 0.99 |
| **3** | 464 | 0.99 | 0.98 | 0.99 | 0.99 | **46** | 1,021 | 0.96 | 0.96 | 0.96 | 0.96 |
| **4** | 404 | 0.97 | 0.96 | 0.96 | 0.96 | **47** | 60 | 0.98 | 0.98 | 0.98 | 0.98 |
| **5** | 517 | 0.98 | 0.98 | 0.98 | 0.98 | **48** | 109 | 0.92 | 0.91 | 0.91 | 0.91 |
| **6** | 331 | 0.91 | 0.90 | 0.90 | 0.90 | **49** | 1,076 | 0.94 | 0.93 | 0.93 | 0.93 |
| **7** | 58 | 0.63 | 0.55 | 0.60 | 0.60 | **50** | 326 | 0.93 | 0.93 | 0.93 | 0.93 |
| **8** | 534 | 0.67 | 0.56 | 0.60 | 0.60 | **51** | 480 | 0.85 | 0.83 | 0.86 | 0.86 |
| **9** | 650 | 0.94 | 0.94 | 0.94 | 0.94 | **52** | 605 | 0.93 | 0.90 | 0.91 | 0.91 |
| **10** | 367 | 0.95 | 0.95 | 0.95 | 0.95 | **53** | 193 | 0.92 | 0.92 | 0.92 | 0.92 |
| **11** | 148 | 0.93 | 0.91 | 0.91 | 0.91 | **54** | 172 | 0.88 | 0.87 | 0.87 | 0.87 |
| **12** | 686 | 0.96 | 0.96 | 0.96 | 0.96 | **55** | 771 | 0.93 | 0.93 | 0.93 | 0.93 |
| **13** | 1,777 | 0.97 | 0.97 | 0.97 | 0.97 | **56** | 562 | 0.87 | 0.87 | 0.88 | 0.88 |
| **14** | 302 | 0.93 | 0.92 | 0.92 | 0.92 | **57** | 102 | 0.98 | 0.98 | 0.98 | 0.98 |
| **15** | 42 | 0.96 | 0.95 | 0.95 | 0.95 | **58** | 435 | 0.98 | 0.98 | 0.98 | 0.98 |
| **16** | 106 | 0.98 | 0.98 | 0.98 | 0.98 | **59** | 91 | 0.95 | 0.94 | 0.95 | 0.95 |
| **17** | 914 | 0.99 | 0.99 | 0.99 | 0.99 | **60** | 906 | 0.95 | 0.95 | 0.95 | 0.95 |
| **18** | 424 | 0.94 | 0.94 | 0.94 | 0.94 | **61** | 1,133 | 0.89 | 0.89 | 0.90 | 0.90 |
| **19** | 744 | 0.91 | 0.90 | 0.90 | 0.90 | **62** | 822 | 1.00 | 0.99 | 0.99 | 0.99 |
| **20** | 876 | 0.85 | 0.84 | 0.85 | 0.85 | **63** | 239 | 0.94 | 0.94 | 0.94 | 0.94 |
| **21** | 328 | 0.96 | 0.95 | 0.95 | 0.95 | **64** | 129 | 1 | 1 | 1 | 1 |
| **22** | 792 | 0.92 | 0.92 | 0.92 | 0.92 | **65** | 577 | 0.95 | 0.95 | 0.95 | 0.95 |
| **23** | 110 | 0.89 | 0.85 | 0.88 | 0.88 | **66** | 409 | 0.91 | 0.90 | 0.90 | 0.90 |
| **24** | 1,790 | 0.95 | 0.95 | 0.95 | 0.95 | **67** | 805 | 0.94 | 0.93 | 0.93 | 0.93 |
| **25** | 872 | 0.96 | 0.96 | 0.96 | 0.96 | **68** | 540 | 0.96 | 0.95 | 0.95 | 0.95 |
| **26** | 1,039 | 0.99 | 0.99 | 0.99 | 0.99 | **69** | 749 | 0.79 | 0.76 | 0.75 | 0.75 |
| **27** | 1,529 | 0.78 | 0.75 | 0.77 | 0.77 | **70** | 1,094 | 0.91 | 0.91 | 0.91 | 0.91 |
| **28** | 983 | 0.69 | 0.51 | 0.61 | 0.61 | **71** | 168 | 0.90 | 0.83 | 0.83 | 0.83 |
| **29** | 407 | 0.97 | 0.97 | 0.97 | 0.97 | **72** | 710 | 0.98 | 0.98 | 0.98 | 0.98 |
| **30** | 858 | 0.95 | 0.95 | 0.95 | 0.95 | **73** | 225 | 0.87 | 0.87 | 0.87 | 0.87 |
| **31** | 53 | 0.96 | 0.96 | 0.96 | 0.96 | **74** | 693 | 0.89 | 0.89 | 0.89 | 0.89 |
| **32** | 827 | 0.93 | 0.93 | 0.94 | 0.94 | **75** | 262 | 0.95 | 0.94 | 0.95 | 0.95 |
| **33** | 868 | 0.96 | 0.95 | 0.95 | 0.95 | **76** | 135 | 0.60 | 0.40 | 0.42 | 0.42 |
| **34** | 142 | 0.99 | 0.99 | 0.99 | 0.99 | **77** | 207 | 0.81 | 0.77 | 0.77 | 0.77 |
| **35** | 683 | 0.93 | 0.92 | 0.92 | 0.92 | **78** | 218 | 0.94 | 0.93 | 0.93 | 0.93 |
| **36** | 73 | 0.95 | 0.95 | 0.95 | 0.95 | **79** | 536 | 0.77 | 0.75 | 0.76 | 0.76 |
| **37** | 185 | 0.95 | 0.95 | 0.95 | 0.95 | **80** | 305 | 0.94 | 0.94 | 0.94 | 0.94 |
| **38** | 823 | 0.95 | 0.95 | 0.95 | 0.95 | **81** | 1,114 | 0.95 | 0.95 | 0.95 | 0.95 |
| **39** | 755 | 0.89 | 0.89 | 0.90 | 0.90 | **82** | 304 | 0.99 | 0.98 | 0.98 | 0.98 |
| **40** | 479 | 0.90 | 0.90 | 0.91 | 0.91 | **83** | 430 | 0.85 | 0.82 | 0.82 | 0.82 |
| **41** | 940 | 0.95 | 0.95 | 0.95 | 0.95 | **Mean** |  | 0.916 | 0.902 | 0.906 | 0.906 |
| **42** | 664 | 0.94 | 0.94 | 0.94 | 0.94 | **STD** |  | 0.080 | 0.109 | 0.102 | 0.102 |
| **43** | 178 | 0.80 | 0.70 | 0.69 | 0.69 | **SUM** | 45,398 |  |  |  |  |

**Supplementary Table 3:** A gradient-boosting classification algorithm was trained on clustering labels to evaluate whether summary behavioural measures could accurately predict different occupancy clusters. Using leave-one-out group cross-validation, 83 unique models were trained on data from all people living with Alzheimer’s (ADs) in the cohort, except the individual being assessed. The classifier's performance was then evaluated using all nights of data for that individual.

**Supplementary Table 4 – DRI-SI (AD) training performance values.**

| **Dataset** | **Training** | | **Validation** | | **Test** | |
| --- | --- | --- | --- | --- | --- | --- |
| **Model type** | **EBM** | **dummy** | **EBM** | **dummy** | **EBM** | **dummy** |
| **AUC** | 0.9 ± 0.01 | 0.5 ± 0.06 | 0.88 ± 0.01 | 0.5 ± 0.06 | 0.82 ± 0.03 | 0.5 ± 0.07 |
| **DOR** | 19.6 ± 2.09 | 1.06 ± 0.36 | 15.41 ± 2.24 | 1.07 ± 0.37 | 8.9 ± 3.11 | 1.1 ± 0.48 |
| **f1_score** | 0.82 ± 0.01 | 0.5 ± 0.05 | 0.8 ± 0.01 | 0.5 ± 0.05 | 0.73 ± 0.05 | 0.5 ± 0.07 |
| **MCC** | 0.63 ± 0.02 | 0.0 ± 0.08 | 0.59 ± 0.02 | 0.0 ± 0.09 | 0.48 ± 0.07 | 0.0 ± 0.1 |
| **Negative precision** | 0.82 ± 0.01 | 0.5 ± 0.04 | 0.8 ± 0.01 | 0.5 ± 0.04 | 0.73 ± 0.05 | 0.5 ± 0.05 |
| **Precision** | 0.81 ± 0.01 | 0.5 ± 0.04 | 0.79 ± 0.01 | 0.5 ± 0.04 | 0.76 ± 0.02 | 0.5 ± 0.05 |
| **Sensitivity** | 0.83 ± 0.01 | 0.5 ± 0.06 | 0.81 ± 0.02 | 0.5 ± 0.06 | 0.7 ± 0.07 | 0.5 ± 0.09 |
| **Specificity** | 0.8 ± 0.01 | 0.5 ± 0.06 | 0.78 ± 0.02 | 0.5 ± 0.06 | 0.77 ± 0.02 | 0.5 ± 0.06 |

**Supplementary Table 4 :** Performance Metrics for the Training, Validation, and Internal Test Sets. Performance metrics of the Dementia Research Institute Sleep Index (DRI-SI (AD)) model using the Explainable Boosting Machine (EBM) compared to a dummy model. The metrics are calculated for the training set, validation set, and internal test set. Each metric is reported with its mean ± standard deviation across 1000 iterations. Model Type: Indicates whether the performance metrics are for the Explainable Boosting Machine (EBM) or the dummy model. AUC: Area Under the Receiver Operating Characteristic Curve. A higher AUC indicates better model performance. DOR: Diagnostic Odds Ratio. Higher values indicate better discriminative performance. F1 Score: The harmonic mean of precision and recall, providing a single metric to evaluate the balance between the two. MCC: Matthews Correlation Coefficient. A measure of the quality of binary classifications, ranging from -1 to 1, where 1 indicates perfect prediction. Neg Precision: Precision for the negative class. The ratio of true negative predictions to the total number of negative predictions. Pos Precision: Precision for the positive class. The ratio of true positive predictions to the total number of positive predictions. Sensitivity: Also known as recall. The ratio of true positive predictions to the total number of actual positives. Specificity: The ratio of true negative predictions to the total number of actual negatives.
